# A Crowdsourcing Approach to Developing and Assessing Prediction Algorithms for AML Prognosis

**DOI:** 10.1371/journal.pcbi.1004890

**Published:** 2016-06-28

**Authors:** David P. Noren, Byron L. Long, Raquel Norel, Kahn Rrhissorrakrai, Kenneth Hess, Chenyue Wendy Hu, Alex J. Bisberg, Andre Schultz, Erik Engquist, Li Liu, Xihui Lin, Gregory M. Chen, Honglei Xie, Geoffrey A. M. Hunter, Paul C. Boutros, Oleg Stepanov, DREAM 9 AML-OPC Consortium, Thea Norman, Stephen H. Friend, Gustavo Stolovitzky, Steven Kornblau, Amina A. Qutub

**Affiliations:** 1 Rice University, Houston, Texas, United States of America; 2 IBM Computational Biology Center, Yorktown Heights, New York, United States of America; 3 The University of Texas MD Anderson Cancer Center, Houston, Texas, United States of America; 4 Arizona State University, Tempe, Arizona, United States of America; 5 Ontario Institute for Cancer Research, Toronto, Ontario, Canada; 6 Department of Medical Biophysics, University of Toronto, Toronto, Canada; 7 Institute for Systems Biology, Moscow, Russia; 8 Sage Bionetworks, Seattle, Washington, United States of America; 9 Department of Genetics and Genomics Sciences, Icahn School of Medicine at Mount Sinai, New York, New York, United States of America; University of Pennsylvania, UNITED STATES

## Abstract

Acute Myeloid Leukemia (AML) is a fatal hematological cancer. The genetic abnormalities underlying AML are extremely heterogeneous among patients, making prognosis and treatment selection very difficult. While clinical proteomics data has the potential to improve prognosis accuracy, thus far, the quantitative means to do so have yet to be developed. Here we report the results and insights gained from the DREAM 9 Acute Myeloid Prediction Outcome Prediction Challenge (AML-OPC), a crowdsourcing effort designed to promote the development of quantitative methods for AML prognosis prediction. We identify the most accurate and robust models in predicting patient response to therapy, remission duration, and overall survival. We further investigate patient response to therapy, a clinically actionable prediction, and find that patients that are classified as resistant to therapy are harder to predict than responsive patients across the 31 models submitted to the challenge. The top two performing models, which held a high sensitivity to these patients, substantially utilized the proteomics data to make predictions. Using these models, we also identify which signaling proteins were useful in predicting patient therapeutic response.

## Author Summary

Acute Myeloid Leukemia (AML) is a hematological cancer with a very low 5-year survival rate. It is a very heterogeneous disease, meaning that the molecular underpinnings that cause AML vary greatly among patients, necessitating the use of precision medicine for treatment. While this personalized approach could be greatly improved by the incorporation of high-throughput proteomics data into AML patient prognosis, the quantitative methods to do so are lacking. We held the DREAM 9 AML Outcome Prediction Challenge to foster support, collaboration, and participation from multiple scientific communities in order to solve this problem. The outcome of the challenge yielded several accurate methods (AUROC >0.78, BAC > 0.69) capable of predicting whether a patient would respond to therapy. Moreover, this study also determined aspects of the methods which enabled accurate predictions, as well as key signaling proteins that were informative to the most accurate models.

## Introduction

AML is a potent malignancy of the bone marrow. It is characterized by the production of dysfunctional myeloid cells, incapable of carrying out their normal differentiation into mature blood cells, ultimately leading to hematopoietic insufficiency, infection, hemorrhage, and anemia [1, 2]. The last decade has seen significant revision in the diagnosis and classification of AML. Classification has shifted from a morphology and lineage centered paradigm, described by the French-American-British (FAB) system, to a system which focuses on genetic anomalies, as described by the new World Health Organization (WHO) guidelines [3]. While this includes many of the genetic mutations now recognized to commonly occur in AML [4], recent sequencing efforts [5] have revealed many previously unrecognized mutations in AML which will require further modification of classification schemes. Moreover, genetic events related to epigenetics and non-coding RNAs have yet to be incorporated into classification. Unfortunately, devising an accurate prognosis for AML patients, particularly those with normal cytogenetics, remains very challenging as the combinatorial potential of genetic events makes for tremendous heterogeneity in both classification and outcome interpretation [6]. This can be attributed, in part, to the fact that only a minority of genetic mutations are driver mutations that lead to functional changes in cellular pathways that translate into physiological outcomes.

High-throughput proteomics studies, such as Reverse Phase Proteomic Arrays (RPPA), have the potential to bridge the gap between the underlying genetic alterations and functional cellular changes. Thus far, proteomics has been used successfully to profile AML patients based on alterations in several key signaling pathways, including highly implicated proteins like FLi1 [7] and FOXO3A [8]. However, these studies also confirm that AML remains a very heterogeneous disease, even on the level of protein signal transduction. It is clear that leveraging high-throughput proteomics to improve the accuracy of prognosis for AML patients will require the development of robust quantitative tools. To date, we did not find any studies which address this issue.

The Dialogue for Reverse Engineering Assessment and Methods (DREAM) is a crowdsourcing platform which has accelerated the development of computational tools in the most pertinent areas of biology and medicine, unraveling gene networks [5, 9], predicting drug sensitivity [10], and harnessing predictions to improve prognosis accuracy [11, 12]. Using a challenge based design, DREAM attracts expertise and fosters collaboration across academic fields while providing a mechanism for the robust and unbiased evaluation of computational methods [13–15]. We developed the DREAM Acute Myeloid Leukemia Outcome Prediction Challenge (AML-OPC) following this paradigm.

The DREAM9 AML-OPC was designed to facilitate both the improvement and comprehensive assessment of quantitative AML prognosis methodologies. Challenge participants were provided access to data from 191 AML patients (the training set) seen at the MD Anderson Cancer Center (Houston, TX), while data from an additional 100 AML (the test set) patients was withheld for model evaluation. We chose Response to Therapy (RT) as the primary clinical endpoint because it is a potentially actionable prognosis criterion. However, since a patient’s Remission Duration (RD) and Overall Survival Time (OS) can be informative in planning patient care, these were also included in the challenge objectives.

The DREAM9 AML-OPC included over 270 registered participants and 79 contributing teams, many of which contributed to multiple sub-challenges. Over 60 algorithms were contributed, many of which were refined during the challenge, yielding several innovative and accurate top performing models. We identify these models, test them for robustness, and determine which scoring metrics differentiate the top performers. We also evaluate whether prediction accuracy can be improved by aggregating predictions from the many diverse models we tested. In addition, we evaluate RT predictions over the population of models to determine which outcomes are more difficult to predict accurately. Finally, we investigate the top two performing models to determine the extent their RT predictions were improved by the RPPA data.

## Results

### Design and implementation of the DREAM 9 AML-OPC

**Fig 1:**
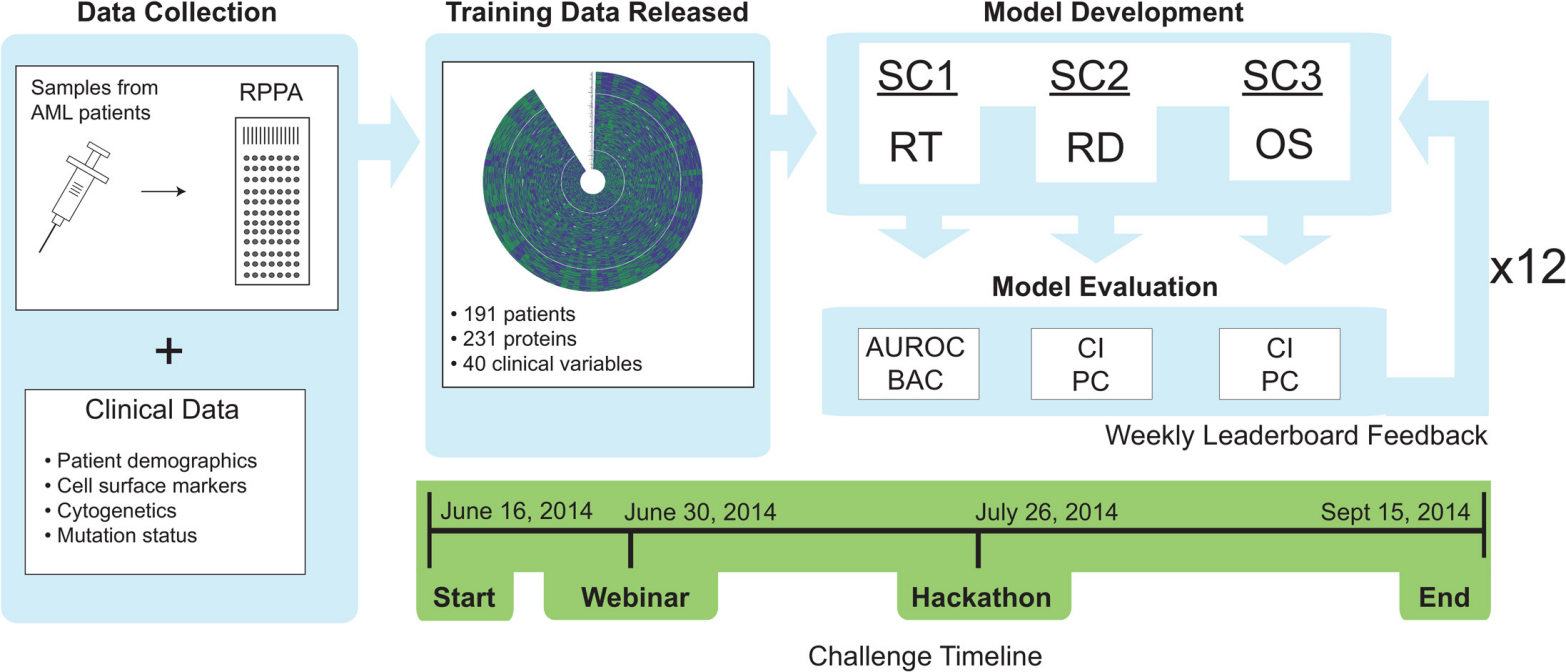
Overview of the DREAM9 AML-OPC data, implementation, and timeline. Samples were collected from 291 patients diagnosed with AML and the levels of 231 signaling proteins were assayed using RPPA. Data was selected from 191 of these patients and released to participants on June 16^th^ 2014 for model training. DREAM9 AML-OPC consisted of 3 subchallenges (SC1, SC2 and SC3) which were evaluated independently on a set of 100 patients (the evaluation was performed on different subsets of 70 patients every week), asking participants to predict either Response to Therapy (RT), Remission Duration (RD), or Overall Survival Time (OS). Participants were given weekly feedback on model performance, which was evaluated using two different metrics for each subchallenge, until September 15^th^ 2014 when the challenge concluded. The metrics were Area Under the Receiver Operating Characteristic (AUROC) curve and Balanced Accuracy (BAC) for SC1, and Concordance Index (CI) and Pearson Correlation (PC) for SC2 and SC3. A hackathon was organized during the Challenge to foster collaboration between participants

The challenge data consisted of 40 clinical indicators (see S1 Table) and 231 RPPA measurements (Fig 1). Three separate sub-challenges were defined to independently address each pertinent aspect of AML prognosis, namely RT for sub-challenge 1 (SC1), RD for sub-challenge 2 (SC2), and the OS for sub-challenge 3 (SC3) (Fig 1). Two metrics were used to evaluate the performance of models within each sub-challenge. In SC1, RT predictions were contributed as list of confidences indicating the probability that each patient would respond to therapy. The area under the receiver operating characteristic (AUROC) and balanced accuracy (BAC, defined as the average of true positive rate and true negative rate) were selected to assess the RT predictions given their wide use and well documented utility in evaluating classification problems. For SC2 and SC3, RD and OS predictions were submitted as a list of remission or survival times (weeks), respectively, along with a list of corresponding prediction confidences. Both SC2 and SC3 were assessed using the concordance index (CI), which evaluates the ranks of predicted versus actual times when there is censored data and is commonly used in survival analysis. Since the CI considers only the order but not the actual values of the predictions, the Pearson correlation (PC) was also used to evaluate RT and OS.

### Evaluating individual and aggregate model performance

**Fig 2:**
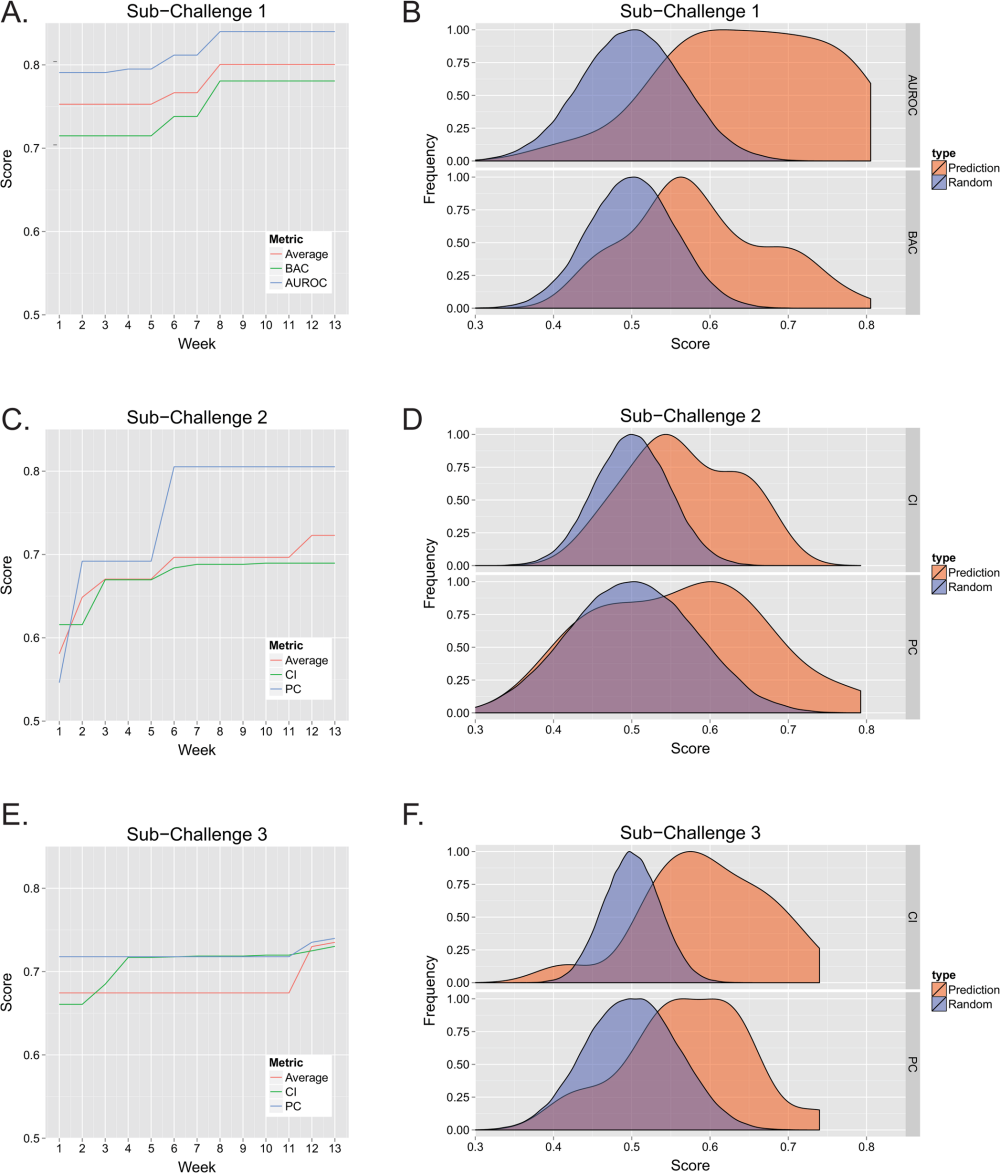
Model performance. The performance of each model was tracked during each week of the challenge. Each sub-challenge was scored using two different metrics. BAC and AUROC were used for SC1, while CI and PC were chosen for SC2 and SC3. The score of the highest performing model was determined each week, either using each metric independently, or by averaging both metrics, and is shown for SC1 (A), SC2 (B), and SC3 (C). Note, if the highest score for any week did not exceed the previous weeks score, the previous score was maintained. The probability density of the final scores (normalized to a maximum of 1) was also determined and for each metric in SC1 (B), SC2 (D), and SC3 (F). The probability density of the null hypothesis, determined by scoring random predictions, is also indicated.

The number of teams contributing model predictions increased for each sub-challenge throughout the DREAM9 AML-OPC (S1A Fig). Participants were allowed to test predictions once per week for a total of 12 weeks (Fig 2). The same test set was used in the leaderboard phase as well as in the final evaluation. Therefore, predictions were scored on a different subsampled (~75%) subset of the 100 patient test set each week to avoid over-fitting. See Materials and Methods for a more detailed description of the challenge design. Final predictions were collected on the 13^th^ week following the challenge opening. In SC1 (Fig 2A), the difference in performance between the top RT predictions from the first week and that from the best performing predictions observed during any week of the competition was an increase of 6.21% when evaluated by the AUROC metric alone, 9.20% when evaluated by the BAC alone, and 6.33% when calculating the best average of the two metrics scored by any model. Here, we used the average of both metrics as a summary statistic for the two metrics. The maximum performance observed during individual weeks is shown in S1B–S1D Fig (red line). The performance of predictions submitted for the final scoring (week 13) were distributed in a manner distinct from random predictions (see Fig 2B, p< 0.01 for AUROC and BAC, Wilcoxon rank sum test), with the top scores being significantly better than random. Note, the median score for each of the previous weeks was also consistently higher than that associated with random predictions (S1B Fig). The scores from predictions made on the final submission test data (week 13) were frequently lower compared to those made on the training data (S2 Fig), particularly for the lower ranked models, suggesting that over-fitting was an important factor in determining model performance. For SC1, the top-performing model used a novel evolutionary weighting approach to feature selection (see S1 Text), yielding a final AUROC score of 0.796 and a BAC of 0.779.

The initial performance of models in predicting RD in SC2 was much lower than observed for RT in SC1, revealing RD predictions were considerably more challenging (Fig 2C). Even so, generous improvement was seen in both the peak PC and CI scores when comparing the initial scores to the best score observed during the challenge, 47.43% and 11.99% respectively. The highest average metric scores observed during the challenge also showed a marked increase (24.43%). While the distributions of CI and PC scores in the final submission were not as separated from random as the RT predictions (p<0.01 for CI, p<0.025 for PC, Wilcoxon rank sum test) (Fig 2D), the top scores were higher than expected for random predictions. With the exception of the PC metric in the first week, median scores were higher than expected for random predictions (S1C Fig). In SC3, OS predictions showed significant improvement when assessing by the CI alone (10.53%), however, the PC showed less increase (~3%) (Fig 2E). The top average of both metrics showed significant improvement (8.99%) as well. The OS final CI and PC predictions were both significantly shifted from random (p<0.01, Wilcoxon rank sum test) (Fig 2F). The top performing approach for both SC2 and SC3 was developed by a single team and based on Cox Regression (see supplemental text). The model achieved final CI and PC scores of 0.655 and 0.773 for RD predictions in SC2, while obtaining scores of 0.730 and 0.740 for SC3.

**Fig 3:**
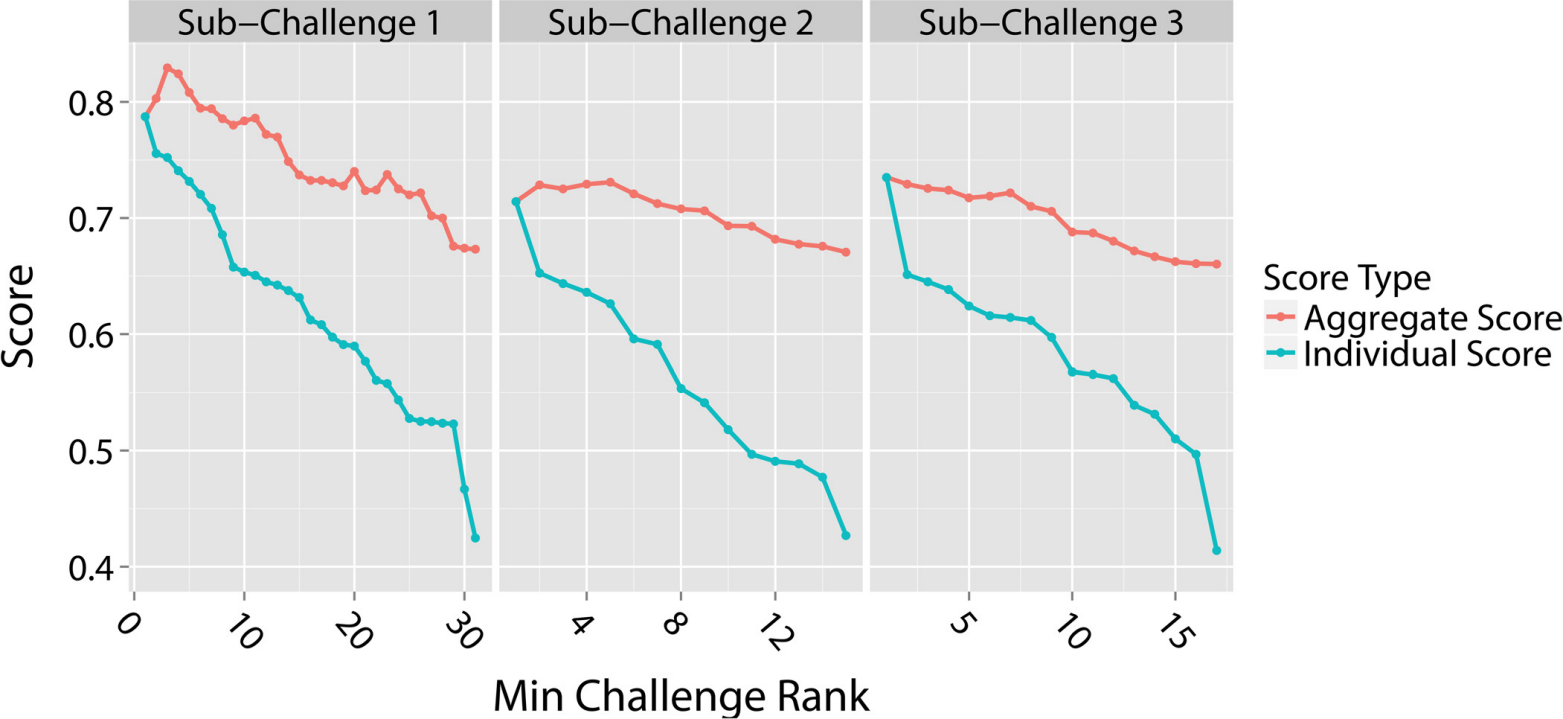
Aggregate and individual model scores. Aggregate scores were determined by averaging the predictions of each model with the predictions from all the models that out-performed it. Model rank is plotted along the x-axis from highest to lowest, with a rank of 1 assigned to the top performing team. Therefore, any given point along the x-axis indicates the minimum rank of the model included in the aggregate score, e.g., a minimum challenge rank of 2 includes predictions from both the rank 2 team and the rank 1 team which out-performed it. The aggregate scores (red lines) were compared to individual team scores (blue lines) for SC1, SC2, and SC3. In each case, the scores reported are the average of the two metrics used for that sub-challenge.

A unique facet of community based model development is the ability to examine whether the diverse population of submitted models can be combined to either assure or improve predictive power. Previous DREAM challenges have shown that this approach, often referred to as the “wisdom of crowds”, generates ensemble prediction scores that are comparable in performance, and often times better, than the top performing models [16]. This is particularly useful in real situations when we don’t have a gold standard and therefore we are not certain of which one is the top performing model. Here we aggregate model predictions by calculating the arithmetic mean for the predictions of each model and those models with superior performance. These averaged predictions are then scored to determine aggregate model performance. We tested the performance of aggregate predictions for RT in SC1 and found that the performance increased above the top performing model by 0.04 (~5% improvement based on the average of AUROC and BAC scores) when combining predictions for the top 3 models (Fig 3, leftmost panel). The performance remained higher than the top performing model even after combining the top 5 models and only decreased by 0.11 when combining all 31 models. This score, however, was significantly better than the corresponding score of the 31^st^ ranked model (0.67 compared to 0.42).

Similarly, aggregating RD predictions from the top 5 models in SC2 (Fig 3 –middle panel) also increased performance above the top performing model by 0.02. The aggregate score from all 15 model predictions was only 0.04 less than the top performing score but was 0.24 better than the worst performing model (rank 15). While the aggregate score for OS predictions in SC3 was not higher than the top performing model score (Fig 3, rightmost panel), combining all 17 model predictions results in a prediction that is between the best and second best, only reduced the performance by 0.08 with respect to the top performing team, and resulted in an aggregate score that was 0.25 better than the worst performing model.

### Assessing model ranking robustness

**Fig 4:**
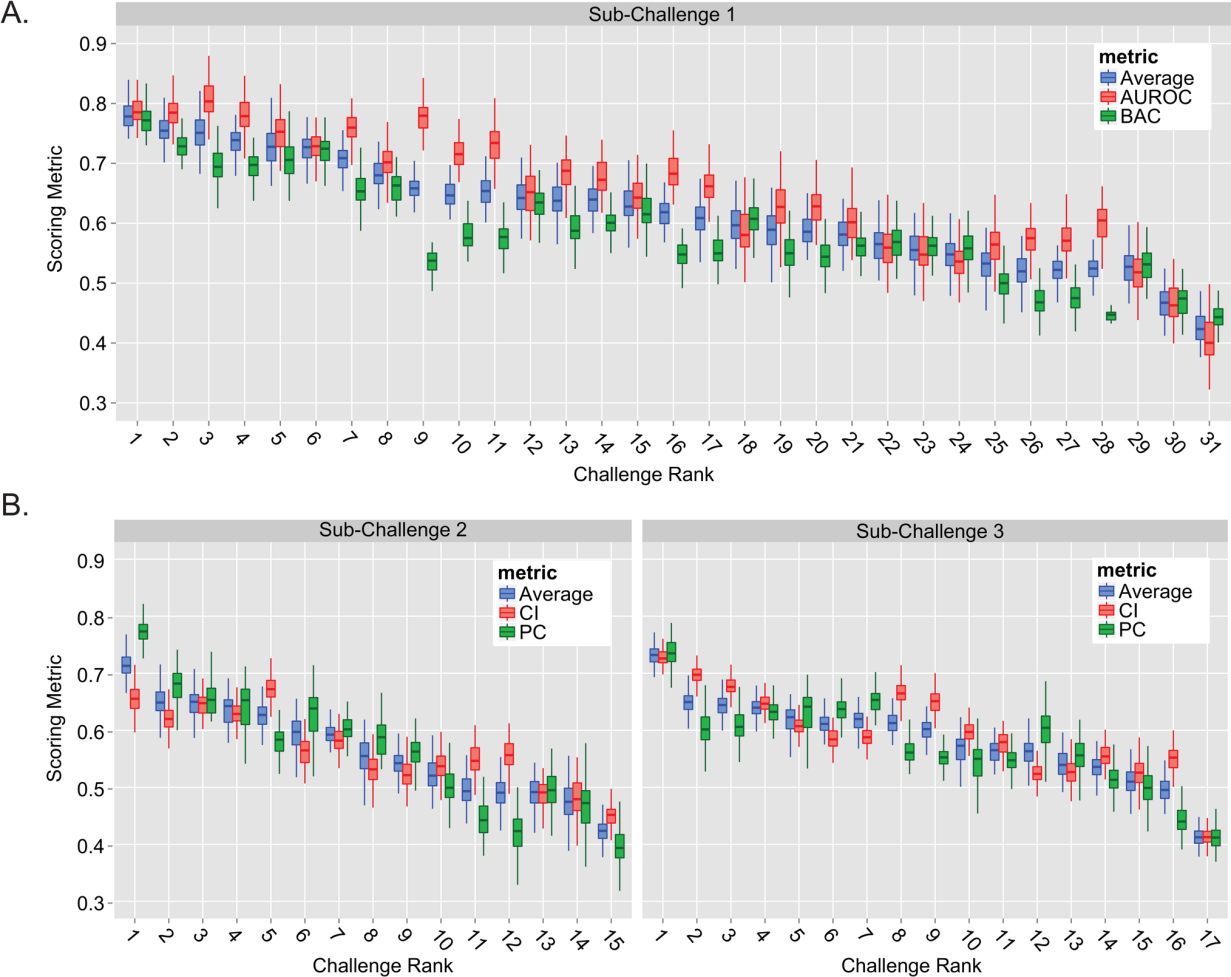
Stability of model performance. Model stability was evaluated for SC1 (A), SC2 (B, left) and SC3 (B, right) by scoring final predictions on 1000 different random subsets of the test set samples (each subset was 60 patients, ~80% of the week 13 test set). The resulting distribution of scores was plotted against each teams overall challenge rank. Note, the center horizontal line of each box indicates the median score. Challenge ranks are ordered from highest to lowest, where a rank of 1 indicates the highest rank.

A key element in assessing model performance is determining the robustness of the final rankings with respect to perturbations of the test set. We evaluated the stability of the final scores by sampling ~81% of the week 13 test set patients (60 patients out of 74), re-scoring each model, and then repeating 1000 times for each sub-challenge (Fig 4). For SC1, the top performing model (Challenge Rank = 1) had a combined metric score that was significantly better than all the lower ranked models (average of AUROC and BAC, Bayes Factor (BF) >6.3 with maximum score overlap of 13.7%, see S3 Fig and Materials and Methods). When examining each metric separately for the top two teams, we found that the distribution of AUROC scores overlapped 33.8% (BF = 1.95), meaning that the BAC set these models apart (overlap of only 3%, BF = 32.3). As indicated earlier, the same model held the best performance in both SC2 and SC3 (Fig 4B, left and right). In SC2, the combined metric score of the top performing model was significantly better than any of the lower ranked models (maximum overlap of 3.1%, BF = 31.3) due to superior performance when evaluated using the PC metric. In contrast, the top model’s resulting CI and PC scores were both superior to the lower ranked models in SC3 (maximum overlap of 3.1%, BF = 31.3).

### The role of patient outcome and proteomics data in determining prediction accuracy

**Fig 5:**
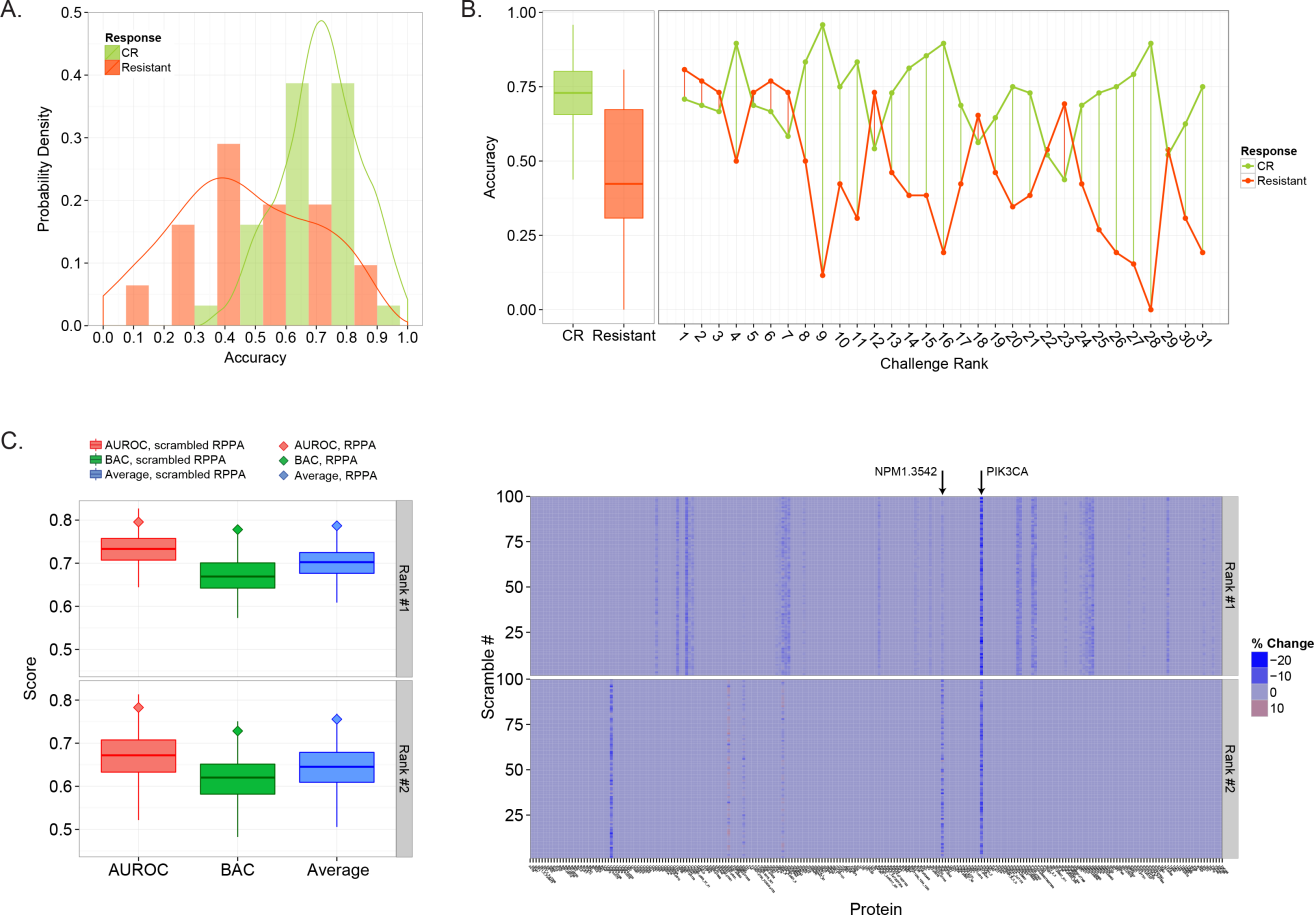
The role of patient outcome and proteomics data in determining prediction accuracy. A) The probability density of prediction accuracy evaluated separately for CR and Resistant patients. (B) Comparison of individual model accuracy for CR and Resistant patients (right) compared to the distribution over the population (left). The midline of the box plot indicated median accuracy while the lower and upper box edge indicated 25^th^ and 75^th^ percentile. (C) The distribution of scores obtained using scrambled RPPA data for the two top performing teams in SC1 (Rank #1 and Rank #2). For each metric, the score obtained using the original RPPA data (not scrambled) is indicated by a diamond. (D) Heat map showing the percent difference in score (average of BAC and AUROC) between predictions obtained using the original RPPA data (not scrambled) and predictions made using data where each protein was scrambled separately over 100 assessments. The y-axis indicates the result for each scrambled protein assessment, 1–100, while the x-axis indicates each protein.

We next investigated prediction errors in more detail, focusing on SC1, since RT is a potentially actionable part of prognosis. Specifically, we asked whether either outcome, Complete Remission (CR) or Resistant, was more difficult to predict. Patients in the test set were grouped based on outcome and the predictions from each model were re-scored. The resulting accuracy, taken as the positive prediction value, was distributed distinctly for each outcome (Fig 5A). The median accuracy for Resistant patients was much lower than CR patients (0.42 vs 0.73, p<0.01, Wilcoxon rank sum test), suggesting they are more difficult to classify (Fig 5B, left). Moreover, 6 of the 7 top performing models achieved accuracies near or above 75% for classifying Resistant patients (Fig 5B, right), well above the median accuracy for that patient group (Fig 5B, left, red box). These same 6 models held accuracies near 70% for CR patients, which were below the median (Fig 5B, left, green box), indicating that accurately classifying Resistant patients set these top models apart. We also examined whether any particular class of learning algorithm was better at predicting the Resistant class of patients, but found a high degree of performance variability amongst implementations that used the same base learners (S4 Fig).

One of the goals of the DREAM9 AML-OPC was to promote the development of a quantitative method which could utilize the high-throughput RPPA proteomics data to make more accurate prognosis predictions. We examined RPPA data usage for the two highest ranked models from SC1. To do so, we tested each model on scrambled RPPA data, meaning the original trends and RPPA data patterns that were present during model training were removed. Note, scrambled protein data was generated by randomly shuffling patient protein values for each individual protein, meaning the distribution and associated statistics were maintained for each protein. Both models were first tested on data with protein values simultaneously scrambled for all 231 proteins a total of 100 times and scored using the AUROC and BAC metrics. Neither model completely lost predictive power, having median scores of 0.69 and 0.65 for the first and second ranked model, respectively, as evaluated using the average of the AUROC and BAC. However, the resulting scores were much lower when the models made predictions using scrambled data compared to the original scores using the actual RPPA data (Fig 5C). For both models, the original scores lay at the upper edge of the distribution of scrambled data scores (top 95%). Using the difference between the original scores and the median scrambled RPPA data scores as an estimate, the performance loss was 0.10 (10.7%) and 0.11 (14.6%) for the top and second ranked model (Fig 5C, compare box midline to diamond for the ‘average’ metric), indicating the RPPA data contributed substantially to each model’s predictions.

We next wanted to determine which specific signaling proteins were most pertinent to the performance of the two top models from SC1. To test this, we scrambled the data for each of the 231 proteins separately over 100 iterations, running each model on a total of 23,100 scrambled data sets. We then evaluated these predictions using a combined metric based on the average of the AUROC and BAC. The percent difference between the original score (unscrambled data) and the score achieved using data with individually scrambled proteins was used to describe the models dependence on each protein (Fig 5D). If a protein was found to influence model performance, data pertaining to that protein was scrambled 10,000 iterations to more accurately assess its impact. For the top performing model (rank #1), randomizing signaling proteins one at a time reduced the model performance in more than 65% of the permutations for 26 proteins (S5A and S5B Fig). For the rank #2 model, 65% or more of the randomizations for each of 4 different proteins decreased model performance (S5C and S5D Fig). Interestingly, perturbing the PIK3CA (Phosphoinositide-3-Kinase, also known as PI3k) signaling protein, an important cell cycle regulator, greatly impacted both models (reducing model performance in more than 96% of the cases, Fig 5D, compare top and bottom heat map, also S5 Fig). Indeed, patients that were classified as resistant to therapy were biased towards low levels (<0) of PIK3CA (chi-squared test, p<0.00018, also see S6 Fig). In addition, the performance of the rank #1 model was also dependent on two other signaling proteins involved in PIK3CA signaling, GSKAB and PTEN. Both models were also dependent on NPM1 (94.36% and 81.43% of permutations reduced performance, rank #1 and rank #2, respectively), a protein which contributes to ribosome assembly and chromatin regulation. Note, both models also utilized several clinical variables (S7 Fig), including Age, Chemotherapy, and AHD.

## Discussion

The absence of new and informative prognostic information has stunted the improvement of AML prognosis accuracy and the advancement of treatment for the last two decades. The DREAM 9 AML-OPC gathered researchers from all around the world to address this problem, successfully providing a competitive incentive for progress while maintaining a collaborative environment. This was evident from both the improvement seen in the challenge leaderboards and the wide use of the challenge forums during the competition to convey ideas and voice questions and concerns. In addition, the DREAM9 AML-OPC carried out a webcast “hackathon”, a collaborative tool new to DREAM challenges, where several teams shared insights and local experts presented ideas.

By evaluating the predictions from both good and poor performing models, we were able to use the DREAM9 AML-OPC as a crowdsourcing platform to gain general insight into making more accurate RT predictions. Although many of the models in SC1 were robust, we determined that higher ranked models were distinct in having an elevated and stable median BAC score. In this case, it is likely that the AUROC metric was less sensitive to the class imbalance inherent in the AML data (as discussed in the methods). As this implies the top performers held greater capacity to predict the minority class, i.e., the Resistant patients, we investigated performance on each class in more detail. Indeed, the overall accuracy observed across all the contributed models was lower in predicting the Resistant cases. The top performing models, however, held accuracies well above the median accuracy for the Resistant class, indicating their ability to predict these patients allowed them to obtain higher BAC scores and higher ranks. Accordingly, future efforts in developing RT prognostic models would benefit from improving predictive ability for Resistant patients.

Each sub-challenge resulted in the development of a refined and robust quantitative method to predict a different aspect of patient prognosis. The top model in SC1 used a random forest learning algorithm coupled with a novel form of feature selection called “evolutionary weighting”. Since no general class of learning algorithms could be identified as more accurate in predicting RT, the success of this algorithm likely stems from its implementation and effective feature selection. While the DREAM 9 AML-OPC focused on clinically actionable RT predictions, the challenge also resulted in the development of a refined Cox regression model capable of predicting RD and OS. In addition, some participants were also inspired to pursue interesting lines of research beyond the specific aims evaluated by the DREAM9-AML-OPC, for example, exploring characteristics specific to subpopulations of patients [17].

It is important to note, however, potential limitations in our challenge design. The scarcity of AML patient proteomics data available required us to use data from the test set to provide participants with feedback on the weekly leaderboard. This represents an indirect form of information leakage which could potentially lead to the development of over-optimistic models. However, we limited feedback to 12 scorings per participant and used random test set subsamples to minimize potential model overfitting. Moreover, the top model from sub-challenge 1 only submitted to the leaderboard 1 time prior to final judging. Another potential source of information leakage was the availability of data describing clinical variables and outcomes for a limited number of patients that were used in this study [18]. This data, however, was released many years prior to the DREAM9 AML-OPC and did not have updated patient outcomes. The proteomics data also originated from a different source, and it does not correlate with the data released for the DREAM9 AML-OPC without informed cross normalization. Therefore, it is unlikely this data would be generally informative if participants decided to use it for model training. As a precaution, data pertaining to these patients was excluded from the final model evaluation (week 13) and therefore did not impact the study results.

Beyond developing accurate prognostic models, participants were provided novel clinical RPPA proteomics data and tasked with developing a means to use this information in conjunction with clinical data to improve prognosis accuracy. To our knowledge, the DREAM9 AML-OPC represents the first attempt at both developing a quantitative means to utilize this information and providing a rigorous way to assess the resulting models. Accordingly, we tested the two top performing models for SC1 to see the extent to which their RT predictions depended on the RPPA data. Our findings indicate that the performance of these models was enhanced by using RPPA data, suggesting that clinical proteomics has the potential to become a valuable component to AML prognosis. Moreover, the performance of both models, though derived from very different approaches, was heavily dependent on PI3KCA, suggesting PI3KCA could be a highly informative protein biomarker for predicting AML patient response to therapy. This is congruent with recent studies suggesting PI3KCA mutation is a prognostic factor for AML [19, 20] and that this protein and pathway is potentially an effective therapeutic target [21]. Both models were also dependent on NPM1. The role of NPM1 mutation as a prognostic factor may be unclear. While it is typically associated with higher survival rates in AML [22, 23], a recent study indicates it is not a prognostic factor for AML patients with normal cytogenetics [24]. Our analysis, based on the performance of predictive models that utilize proteomics data rather than genetic data, indicates that NDM1 is an informative feature in predicting AML patient response to therapy.

## Materials and Methods

### Challenge data

The dataset used for the DREAM 9 AML-OPC consisted of 291 patients seen at the MD Anderson Cancer Center (Houston, TX), for which clinical attributes and RPPA data from bone marrow biopsies was obtained, processed, and normalized as described previously [25–28]. A genetic algorithm was designed to partition the dataset into training and test datasets which have equivalent distributions of clinical and RPPA data. The training set consisted of 191 patients, while the test set held 100 patients. These datasets are available on the Synapse online repository. Note, the clinical outcomes in the overall dataset were imbalanced, with the percent of CR and Resistant patients being approximately 71% and 29% respectively. This ratio was believed to be generally congruent with the overall low survival rate for AML patients and was preserved in both the training and test datasets.

### Challenge implementation

The training data was released to participants on June 16^th^, 2014. Participants were allowed to submit test set predictions for feedback once a week for 13 weeks, from June 23^rd^ to September 8^th^, 2014 (see Fig 1 for timeline). For each sub-challenge, models were evaluated using two different metrics, and the values for these metrics were posted to the leaderboard each week. Metrics were the AUROC and BAC for SC1, and the CI and PC for both SC2 and SC3. To prevent model over-fitting, 75 out of 100 patients were selected at random for scoring for weeks 1–11. For weeks 12 and 13, 74 patients were selected to exclude patients for which limited amounts of data might have been available from other sources. Note, SC2 and SC3 required censoring of patients for the purposes of scoring. In SC2, the PC was calculated for RD predictions based solely on patients that responded to therapy and underwent a subsequent relapse. Likewise, for OS predictions in SC3, the PC was determined only for patients that were known to be deceased. For both SC2 and SC3, the CI was determined using right censoring. Final submissions were taken on September 15^th^, 2014 and scored as described above.

Part of the challenge design included fostering collaboration amongst participants. During the challenge, model scores were posted on a weekly leaderboard so that the progress of every participant was shared throughout the DREAM community. An open “Hackathon” took place on July 26^th^ as part of an effort to foster collaboration in the challenge community. In addition, a community forum was set up so registered participants could ask both technical and administrative questions about the challenge, share ideas, and voice concerns.

### Robustness analysis

To check if the ranking resulting from the final model predictions is robust to perturbations of the test set (e.g., removing some of the patients), we re-evaluated each model’s predictions on 1000 sub-samples of the final (week13) test patients. The results of the performance comparison between the model ranked 1^st^ (Rank #1) using the final test set and the models ranked 2^nd^, 3^rd^, etc (Rank #2, Rank #3, etc) are shown in S3 Fig. More precisely, if we call ΔM_1k_ the difference in performance metrics of the Rank #1 model (M_1_) and model Rank k (M_k_), then ΔM_1k_ = M_1_—M_k_, under the same sub-sample. S3 Fig shows the distribution of values of ΔM_1k_ as a function of k. For SC1, the Rank #2 model scores better than Rank #1 in the averaged AUROC and BAC score (that is, ΔM_12_ is negative) in 13.7% of the sub-samples tested. Therefore, while the Rank #1 model does not perform better than the Rank #2 model in all sub-samples, it scores higher with a frequency of 86.3%. If we call Prob(M_1_ > M_k_ | D) the probability that Model Rank#1 scores higher than model Rank k, and Prob(M_k_ > M_1_ | D) the probability that model Rank k scores better than model Rank #1 given the data, then the posterior odd ratio is defined as:

$${\text{O}}^{\text{post}}\left(1,\text{k}\right)=\text{Prob}({\text{M}}_{1}>{\text{M}}_{\text{k}}\;|\;\text{D})\;/\;\text{Prob}({\text{M}}_{\text{k}}>{\text{M}}_{1}\;|\;\text{D})$$

This ratio measures the fold change of the frequency of model Rank #1 performing higher than model Rank k to the frequency of model Rank k performing better than model Rank #1 given the data at hand. This unprejudiced prior was that mode Rank #1 and model Rank k have equal odds of winning. Therefore the prior odds ratio is given by:

$${\text{O}}^{\text{prior}}\left(1,\text{k}\right)=\text{Prob}({\text{M}}_{1}>{\text{M}}_{\text{k}})\;/\;\text{Prob}({\text{M}}_{\text{k}}>{\text{M}}_{1})=1$$

The Bayes Factor K is defined as the ratio between posterior odds and prior odds ratios:

$$\text{BF}\left(1,\text{k}\right)={\text{O}}^{\text{post}}\left(1,\text{k}\right)/\;{\text{O}}^{\text{prior}}\left(1,\text{k}\right)$$

For hypothesis testing, where the conventional statistical significance is given by p-values < 0.05, well established guidelines for the interpretation of Bayes Factors [29] suggest that BF(1,k) > 3, 20 and 150 gives positive, strong, and very strong evidence in favor of M_1_ > M_k_.

For sub-challenges 1, 2 and 3 we have that BF(1,2) is equal to 6.3, 332 and >999, indicating a robustness of the relative ranking between Rank #1 and Rank #2 models in the Challenge. This robustness holds for all metrics and all sub-challenges, except for metric AUROC in SC1, for which model Rank #1 cannot be considered to be better than model Rank #2, #3 or #4.

### Assessing the importance of the RPPA data

Use of the RPPA data was determined for the two top scoring models in SC1 by scrambling the protein data, making predictions with the previously trained models, and comparing the scores to those from the original predictions that were made with the unscrambled RPPA data. The data was scrambled by randomly shuffling the values for each individual protein across the 100 patients in the test dataset. In this way, the statistical properties, e.g., the mean, variance, range, etc, were preserved for every protein. All proteins in the dataset were scrambled in this manner for each assessment and a total of 100 assessments were conducted. Note, each model was scored using the final (week 13) test dataset (74 patients). Reduction in model performance was measured by the percentage of scores that were lower than the original predictions, i.e, dividing the number of scores that were less than the original (unscrambled) by the total number of scores from scrambled RPPA assessments.

The procedure to determine which specific proteins were informative to the two top performing models was the same as described above, with the exception that only 1 protein was scrambled for each of the assessments. Again, this was repeated 100 times, making a total of 23,100 scrambled assessments for the 231 proteins. To more accurately determine the percentage of perturbations that decreased model performance, an additional 10,000 assessments were performed for proteins that altered model performance under the initial 100 assessments.

### Statistical computing

Challenge results were analyzed using the statistical computing language R [30]. Figure plots were developed using the package ggplot2 [31].

## Supporting Information

S1 Fig Summary of team participation and performance each week. (A) The number of teams participating in each sub-challenge each week. (B-D) Box plots comparing the distribution of scores each week with scores generated from random predictions for each sub-challenge. The red line indicates the maximum score seen for each week.
(TIF)

S2 Fig Comparison of model performance on training and test datasets. Model performance was evaluated on the week 13 test data (red) and training data (blue). (A) Performance for SC1 was determined using the AUROC (top) and BAC (bottom) scores (B) Performance for SC2 was determined using the CI (top) and PC (bottom) scores. (C) Performance for SC3 was determined using the CI (top) and PC (bottom) scores.
(TIF)

S3 Fig Paired differences for stability analysis of model performance. Model stability was evaluated by scoring final predictions on 1000 different random subsets of the week 13 test set patients (81%). For each specific subset, the difference between the Rank #1 model score and each lower ranking model was determined. Positive differences are indicated by blue points while negative differences are shown in red. The text above each set of points indicates the fraction of scores in which a lower ranking model outperformed the rank #1 model.
(TIF)

S4 Fig Accuracy in predicting CR and Resistant patients for different machine learning methods. Each model was classified by its base machine learning method as documented in the write-ups submitted by each participant. The accuracy in predicting both CR and Resistant patients, taken as the positive predictive value, was then determined for each model. Note, “Meta” refers to models that used a combination of multiple different machine learning approaches, while other refers to approaches that did not use machine learning methods. These methods included various implementations of descriptive statistics, probability analysis, and sparse matrix analysis.
(TIF)

S5 Fig Proteins found to impact the performance of the Rank #1 and Rank #2 models in SC1. (A) Box plots comparing the distribution of scores obtained by scrambling individual protein data over 100 assessments (see methods) for the Rank #1 model. Each box centerline indicates the median score while the upper and lower box borders indicate the 25^th^ and 75^th^ percentile respectively. (B) Table showing the percentage of randomizations that yielded reduced scores with respect to the original (unscrambled) RPPA data for the Rank #1 model. (C) Box plots, as described in A, showing the distribution of scores obtained by scrambling individual protein data over 100 assessments for the Rank #2 model. (D) Table showing the percentage of randomizations that yielded reduced scores with respect to the original (unscrambled) RPPA data for the Rank #2 model.
(TIF)

S6 Fig Distribution of PIK3CA levels for patients classified as CR or Resistant. A histogram showing the number of patients for different levels of PIK3CA. The vertical centerline at 0 denotes the boundary between low and high PIK3CA levels.
(TIF)

S7 Fig Clinical variables found to impact the performance of the Rank #1 and Rank #2 models in SC1. (A) Heat map showing the percent difference in score (average of BAC and AUROC) between predictions obtained using the original clinical variables (not scrambled) and predictions made using data where each clinical variable was scrambled separately over 100 assessments. The y-axis indicates the result for each scrambled assessment, 1–100, while the x-axis indicates each clinical variable. (B) Box plots comparing the distribution of scores obtained by scrambling data from individual clinical variables over 100 assessments (see Methods) for the Rank 1 model. Each box centerline indicates the median score while the upper and lower box borders indicate the 25^th^ and 75^th^ percentile respectively. (C) Table showing the percentage of perturbations that resulted in reduced scores after scrambling each individual clinical variable for the Rank #1 model. (D) Box plots, as described in B, showing the distribution of scores obtained by scrambling data pertaining to individual clinical variables over 100 assessments for the Rank #2 model. (E) Table showing the percentage of perturbations that resulted in reduced scores after scrambling each individual clinical variable for the Rank #2 model.
(TIF)

S1 Table Clinical covariates included as data in the DREAM 9 AML-OPC Challenge. (TIF)

S1 Text Participant description of best performing methods. The description of the best performing methods, as provided by participants, for SC1, SC2, and SC3.
(PDF)

S2 Text Membership list for the DREAM 9 AML-OPC consortium. (PDF)
