# Supplementary figures and images for: A Crowdsourcing Approach to Developing and Assessing Prediction Algorithms for AML Prognosis
Source: PLoS Comput Biol. 2016 Jun 28;12(6):e1004890. doi: 10.1371/journal.pcbi.1004890 (PMC4924788; doi:10.1371/journal.pcbi.1004890)

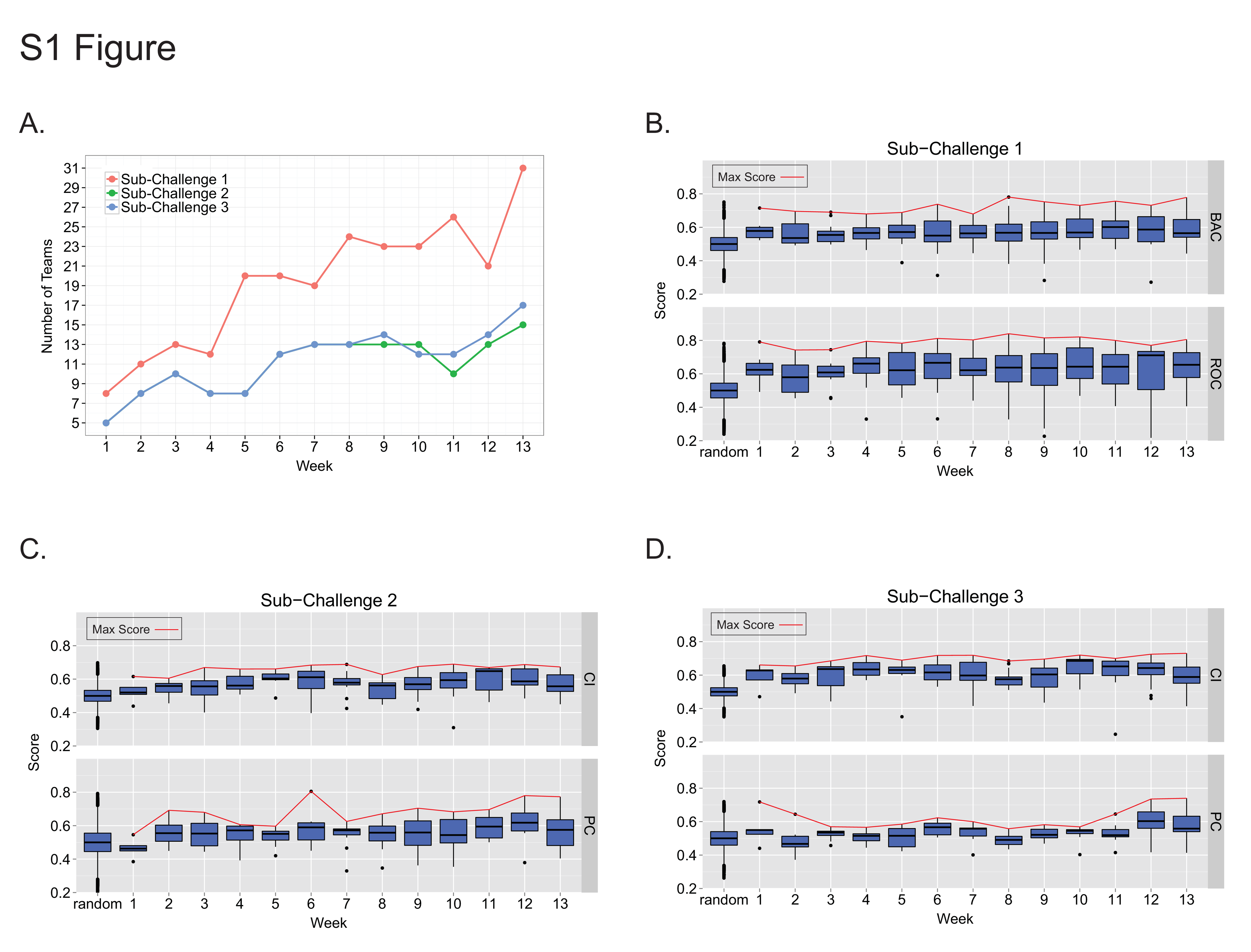

Supplement: S1 Fig — (A) The number of teams participating in each sub-challenge each week. (B-D) Box plots comparing the distribution of scores each week with scores generated from random predictions for each sub-challenge. The red line indicates the maximum score seen for each week. (TIF) [file pcbi.1004890.s001.tif]

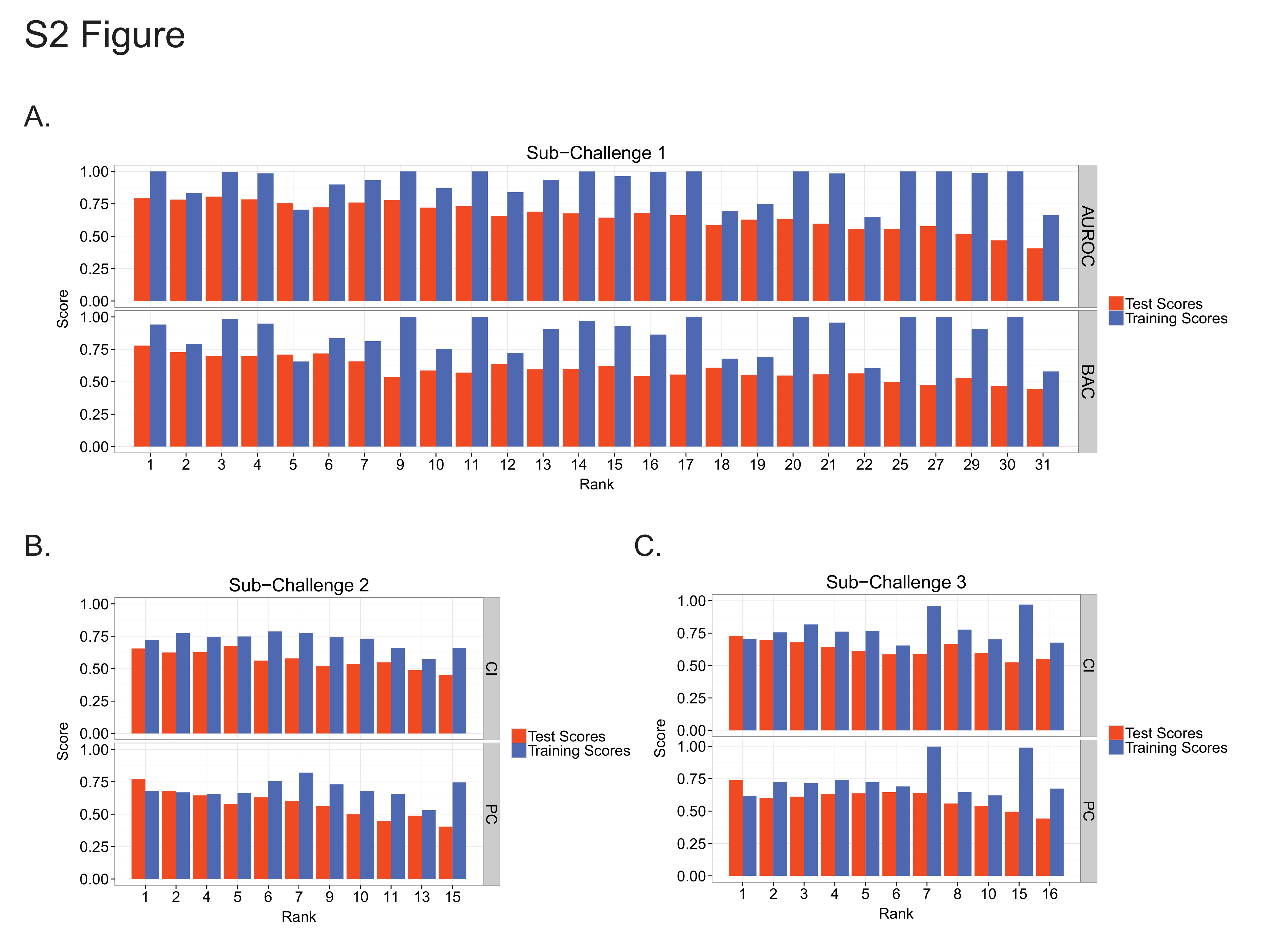

Supplement: S2 Fig — Model performance was evaluated on the week 13 test data (red) and training data (blue). (A) Performance for SC1 was determined using the AUROC (top) and BAC (bottom) scores (B) Performance for SC2 was determined using the CI (top) and PC (bottom) scores. (C) Performance for SC3 was determined using the CI (top) and PC (bottom) scores. (TIF) [file pcbi.1004890.s002.tif]

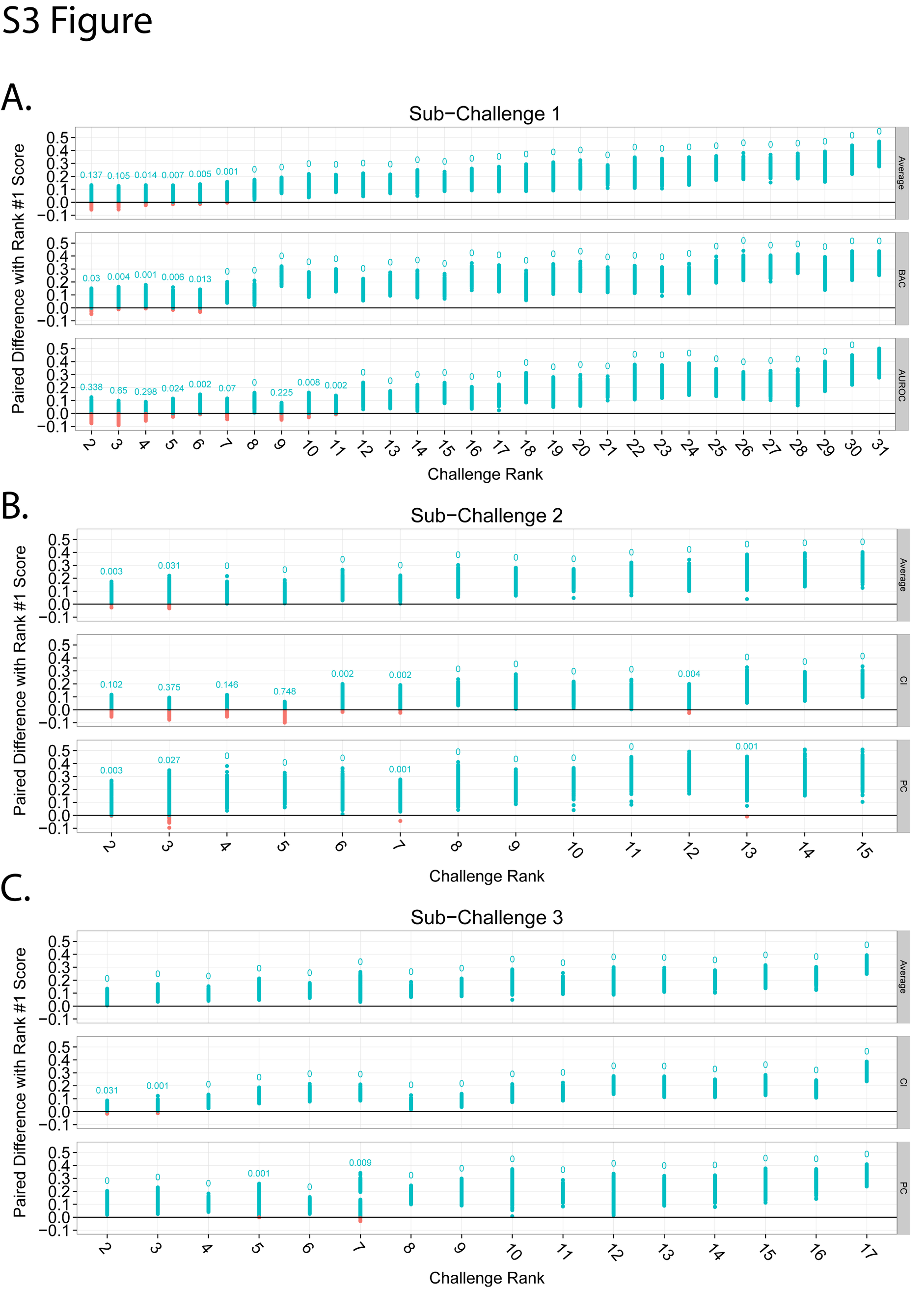

Supplement: S3 Fig — Model stability was evaluated by scoring final predictions on 1000 different random subsets of the week 13 test set patients (81%). For each specific subset, the difference between the Rank #1 model score and each lower ranking model was determined. Positive differences are indicated by blue points while negative differences are shown in red. The text above each set of points indicates the fraction of scores in which a lower ranking model outperformed the rank #1 model. (TIF) [file pcbi.1004890.s003.tif]

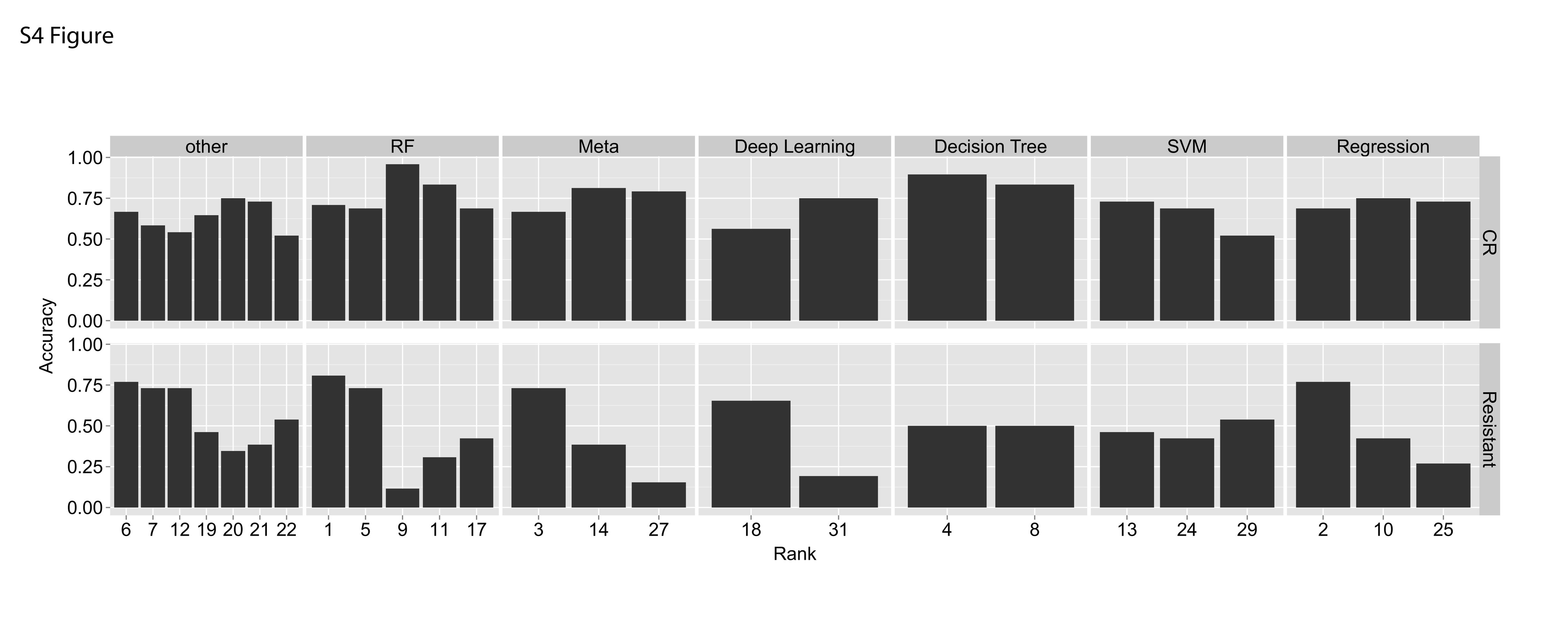

Supplement: S4 Fig — Each model was classified by its base machine learning method as documented in the write-ups submitted by each participant. The accuracy in predicting both CR and Resistant patients, taken as the positive predictive value, was then determined for each model. Note, “Meta” refers to models that used a combination of multiple different machine learning approaches, while other refers to approaches that did not use machine learning methods. These methods included various implementations of descriptive statistics, probability analysis, and sparse matrix analysis. (TIF) [file pcbi.1004890.s004.tif]

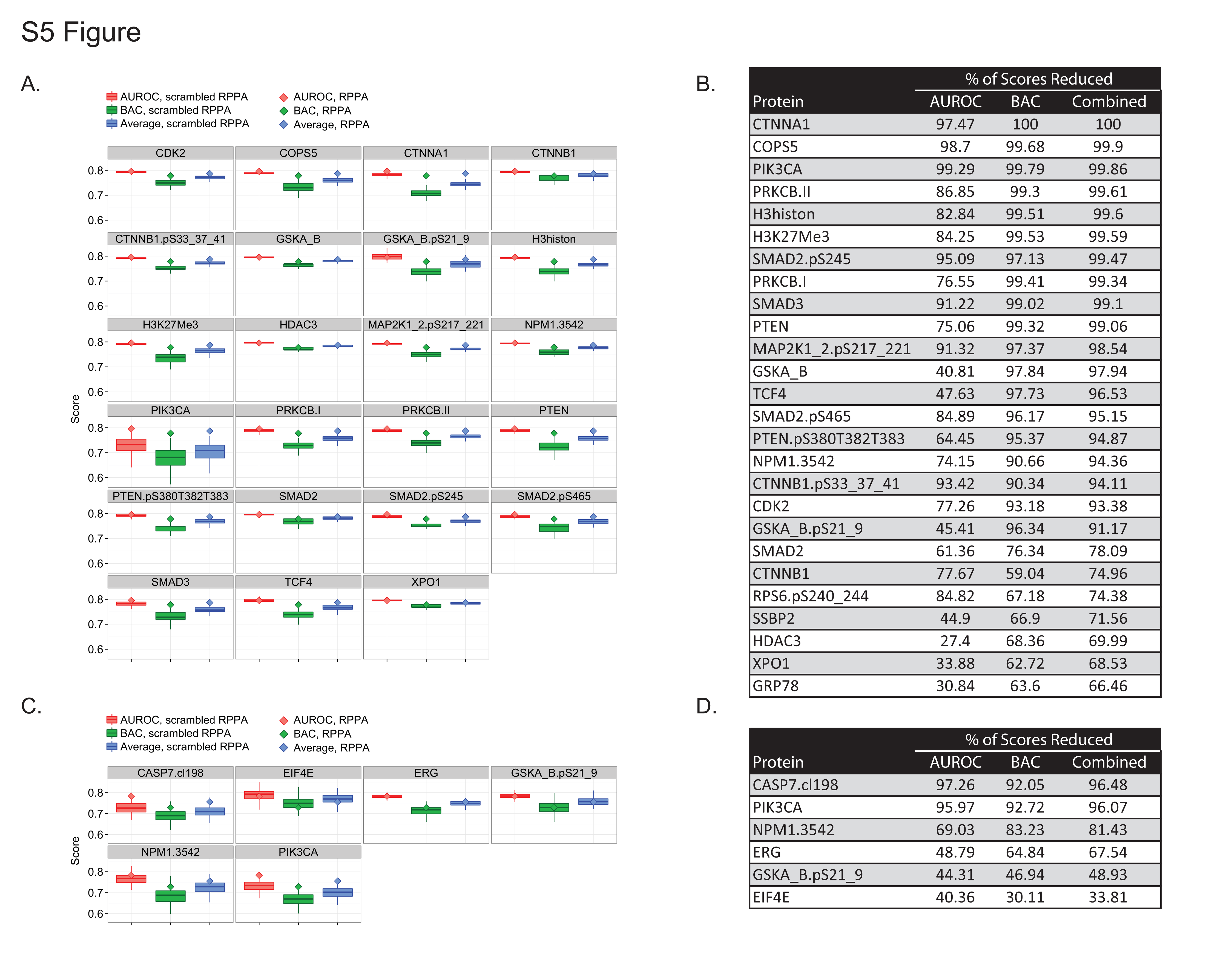

Supplement: S5 Fig — (A) Box plots comparing the distribution of scores obtained by scrambling individual protein data over 100 assessments (see methods) for the Rank #1 model. Each box centerline indicates the median score while the upper and lower box borders indicate the 25th and 75th percentile respectively. (B) Table showing the percentage of randomizations that yielded reduced scores with respect to the original (unscrambled) RPPA data for the Rank #1 model. (C) Box plots, as described in A, showing the distribution of scores obtained by scrambling individual protein data over 100 assessments for the Rank #2 model. (D) Table showing the percentage of randomizations that yielded reduced scores with respect to the original (unscrambled) RPPA data for the Rank #2 model. (TIF) [file pcbi.1004890.s005.tif]

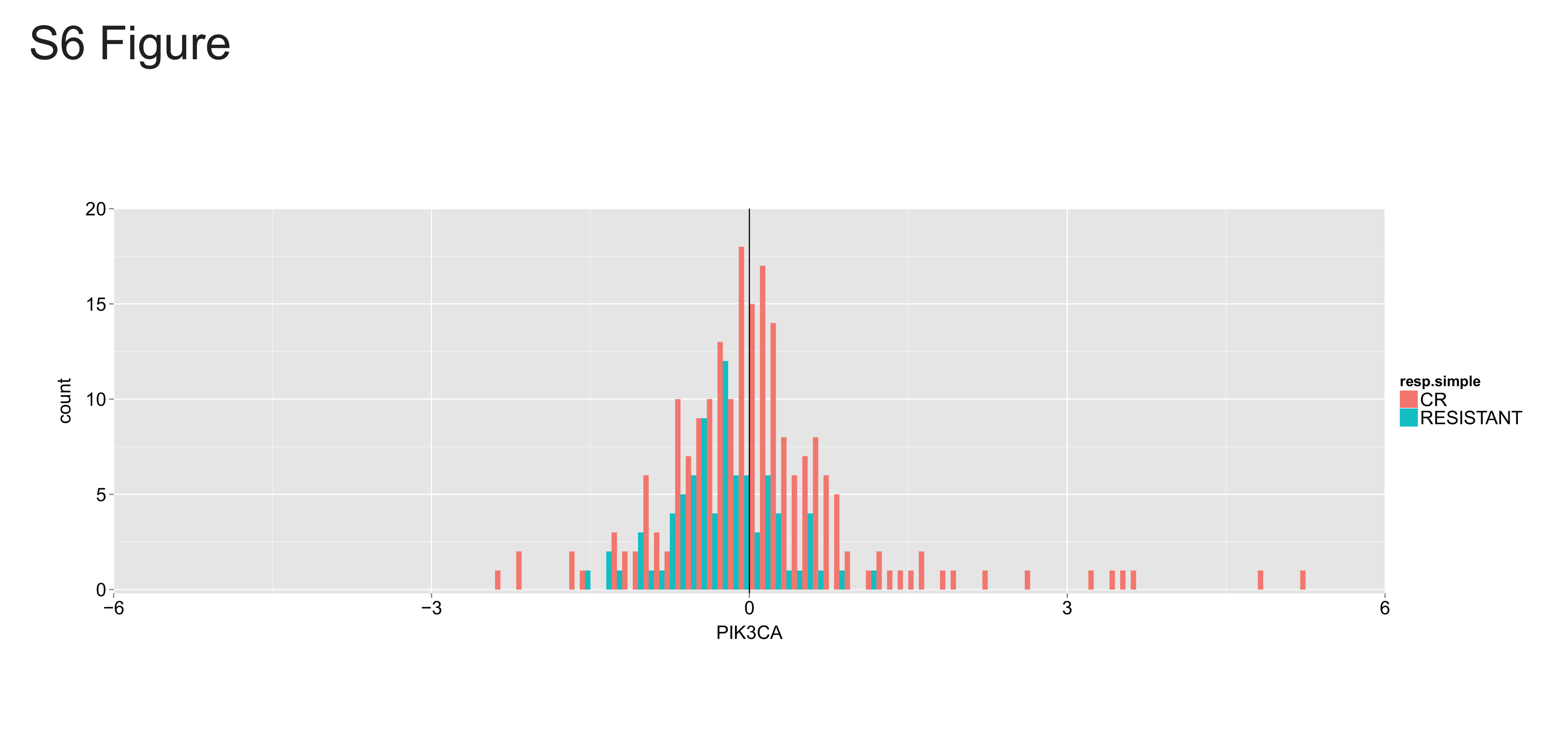

Supplement: S6 Fig — A histogram showing the number of patients for different levels of PIK3CA. The vertical centerline at 0 denotes the boundary between low and high PIK3CA levels. (TIF) [file pcbi.1004890.s006.tif]

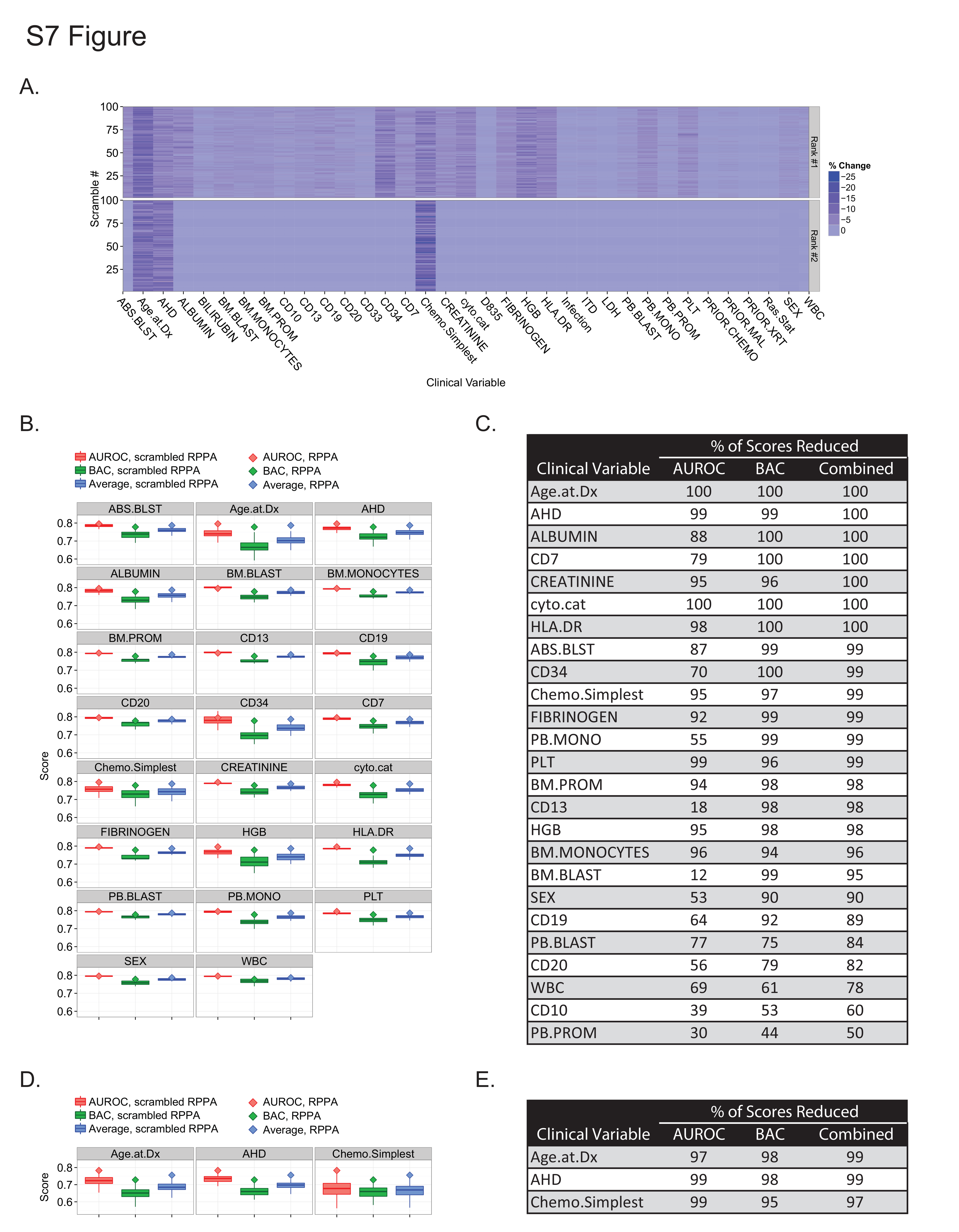

Supplement: S7 Fig — (A) Heat map showing the percent difference in score (average of BAC and AUROC) between predictions obtained using the original clinical variables (not scrambled) and predictions made using data where each clinical variable was scrambled separately over 100 assessments. The y-axis indicates the result for each scrambled assessment, 1–100, while the x-axis indicates each clinical variable. (B) Box plots comparing the distribution of scores obtained by scrambling data from individual clinical variables over 100 assessments (see Methods) for the Rank 1 model. Each box centerline indicates the median score while the upper and lower box borders indicate the 25th and 75th percentile respectively. (C) Table showing the percentage of perturbations that resulted in reduced scores after scrambling each individual clinical variable for the Rank #1 model. (D) Box plots, as described in B, showing the distribution of scores obtained by scrambling data pertaining to individual clinical variables over 100 assessments for the Rank #2 model. (E) Table showing the percentage of perturbations that resulted in reduced scores after scrambling each individual clinical variable for the Rank #2 model. (TIF) [file pcbi.1004890.s007.tif]

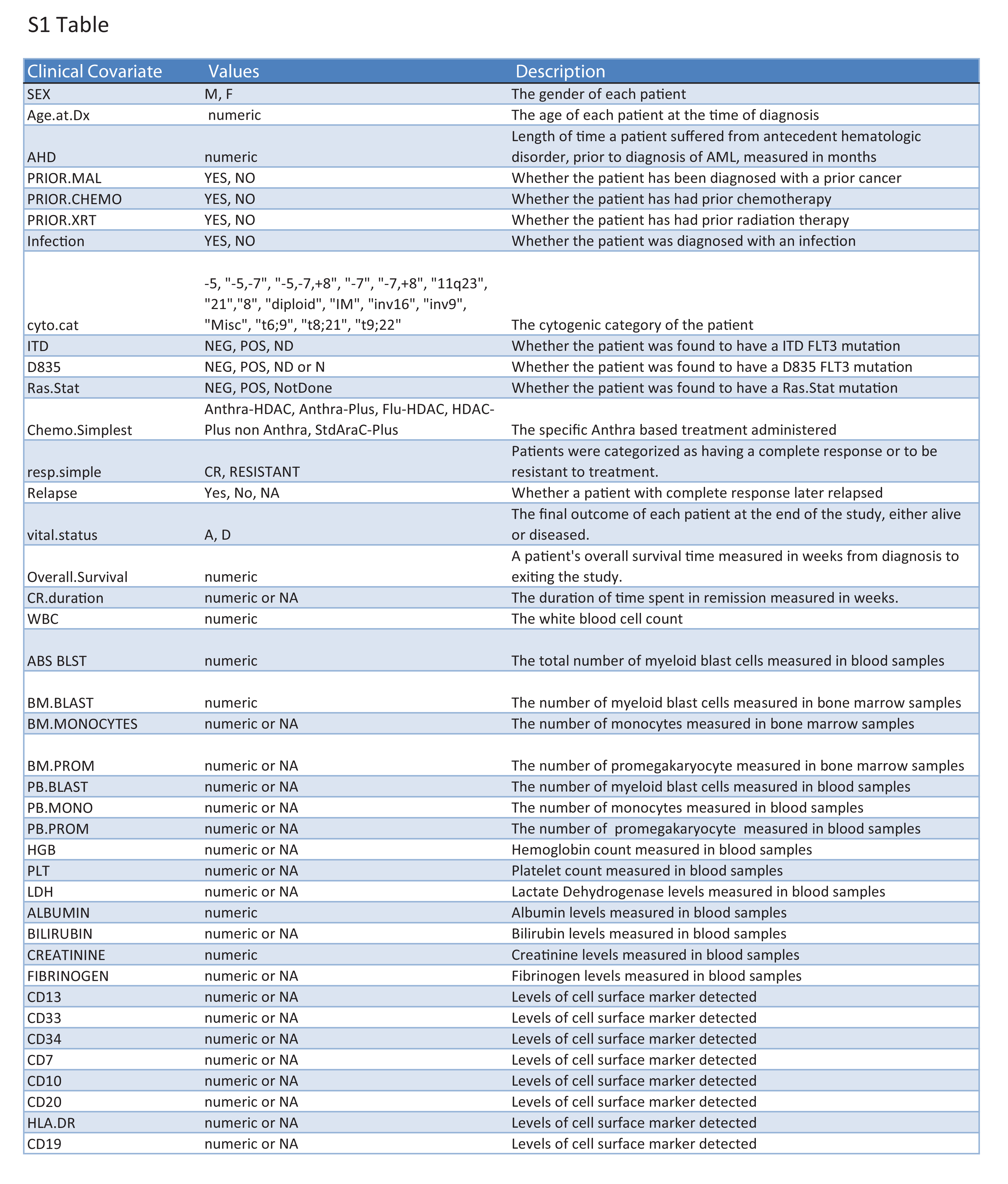

Supplement: S1 Table — (TIF) [file pcbi.1004890.s008.tif]
