# Supplementary material for: A Crowdsourcing Approach to Developing and Assessing Prediction Algorithms for AML Prognosis
Source: PLoS Comput Biol. 2016 Jun 28;12(6):e1004890. doi: 10.1371/journal.pcbi.1004890 (PMC4924788; doi:10.1371/journal.pcbi.1004890)
Supplement: S1 Text — The description of the best performing methods, as provided by participants, for SC1, SC2, and SC3. (PDF) [file pcbi.1004890.s009.pdf]

Sub-challenge #1, Rank #1 Model

Title

Author: Li Liu

Affiliation: Arizona State University

Email: leew369@gmail.com

Summary Sentence

Features are weighted based on their evolutionary conservations and t test p-values during the feature selection step, while un-weighted selected features are used in an ensemble of random forest modeling.

Background/Introduction

From the perspective of long-term evolutionary history, cancer genes are highly conserved among species [1]. In the same vein, cancer driver mutations are almost always found at evolutionarily conserved positions that lead to deleterious loss- or gain- of functions [2]. Based on these observations, it is reasonable to infer that changes of expression levels, another mechanism of functional regulation, will have more profound impact if they involve highly conserved proteins, as compared to less conserved proteins. In fact, studies have shown that expression level is a strong correlate of evolutionary conservation [3]. Therefore, I designed a weighting scheme to improve the RPPA data in predicting AML remissions [4].

The basic idea is to give higher weights to conserved proteins and lower weights to variable proteins. Conservation of a protein can be calculated using evolutionary approaches (see Method). Furthermore, differentially expressed proteins shall be given higher weights, which can be represented by p-values from student t tests. A combination of these two measurements gives rise to the final weights.

After each feature is transformed using its specific weight, feature selection is performed. This process will favor features that are more evolutionarily conserved and more differentially distributed between two classes. In the classification step, models are built upon selected but un-weighted features.

Methods

Preprocessing data: Categorical features were coded using dummy variables. For each feature, the raw values were transformed to z scores, which had mean of 0 and standard deviation of 1. Missing values were imputed by random sampling. Four features with low information content (entropy<0.05) were removed. Since no two features shared > 95% correlation, all of them were treated as independent features.

<u>*Calculating weights*</u>: For each protein, the multiple sequence alignments of 46 vertebrates (Figure 1) were downloaded from the UCSC Genome Browser [5]. At a given position, the evolutionary rate (*r*) is calculated as the number of substitutions per billion years [6]. The conservation of a protein is measured as the average of *r* over all positions. Because high evolutionary rate indicates low conservation, the reciprocal of evolutionary rate (*1/r*) is used as the evolutionary weight (*WE*) for each protein. For clinical features, the evolutionary weights were set to be the maximum of *WE*. For each feature, a two-side student t test was performed. P-values were transformed via negative logarithm (*-log(P)*) and used as the differential weight (*WD*). For each feature *i*, the final weight was the sum of evolutionary and differential weights ($W_i = WE_i + WD_i$).

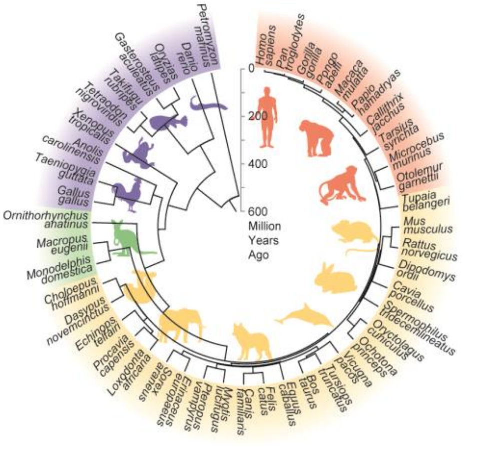

Figure 1. An evolutionary timetree of 46 species used for deriving evolutionary rate for each protein position.

<u>*Constructing ensemble models:*</u> Because the training data are highly unbalanced, we employed an ensemble approach that constructs multiple classification models using balanced subsamples [7]. Specifically, a subset of 50 samples was randomly selected from each class (CR labeled as 1, Resistant labeled as 0). This number was determined as 90% of samples in the under-represented resistant class. In the feature selection step, values of each feature were multiplied by their corresponding weights. Then, stability selection [8] with sparse logistic regression was performed, as implemented in the SLEP package [9]. During stability selection, bootstrapping was used to identify features that were consistently selected among multiple runs with a wide range of regularization parameters. Features identified in >50% of bootstrapping runs were selected. In the classification step, selected features with un-weighed values were used to construct a random forest model with 50 trees. The above procedure was repeated 100 times, which produced an ensemble of 100 random forest models.

<u>*Making predictions:*</u> For each sample in the testing dataset, we derived 100 predictions, one from each random forest model. The confidence score equals the percentage of models that predict the sample as CR.

<u>*Multiple submissions:*</u> In total, I submitted the predictions twice. The first submission was derived from an ensemble of 30 random forest models. The final submission was from 100 models. The correlation between these two submissions was very high (97%). It will be interesting to compare predictions with and without using the weighting scheme. Unfortunately, because I joined the project only two weeks before its closing date, I missed the opportunity to test different models.

Conclusion/Discussion

Because I only have one submission scored (submission ID: 2669636), it is hard to assess the contribution of evolutionary and differential weights to the prediction accuracy. However, this single submission achieved BAC score of 0.7283 and AUROC score of 0.7704, which had an overall rank of #1 in the week of September 8th. Therefore, the performance of this approach is at least comparable to other top-ranked methods.

Meanwhile, I am aware that team YL also participated in the Challenge. The lead of team YL, Dr. Ye, is a close collaborator of mine. In the classification step, both of our teams used an ensemble of random forest models. However, team YL took a different strategy in the feature selection step. Once the models from our two teams are disclosed, we will be able to infer the contribution of evolutionary and differential weights to the prediction accuracy. However, the ultimate evaluation will come from more systematic comparisons.

Author Statement

I thank Dr. Ye for inviting me to the Challenge, and giving advices in using the SLEP package.

References

1. Goymer P. (2007) Genetics: Conserved by evolution, but altered in cancer. *Nature Reviews Cancer.* 7:812-813.
2. Kumar S, Dudley JT, Filipski A, Liu L. (2011) Phylomedicine: an evolutionary telescope to explore and diagnose the universe of disease mutations. *Trends in Genetics.* 27(9):377-86.
3. Drummond DA, Raval A, Wilke CO. (2006) A single determinant dominates the rate of yeast protein evolution. *Molecular Biology and Evolution.* 23(2):327-37.
4. DREAM Acute Myeloid Leukemia Outcome Prediction Challenge (syn2455683)
5. Rosenbloom KR, Sloan CA, Malladi VS, Dreszer TR, Learned K, Kirkup VM, et al. (2013) ENCODE data in the UCSC Genome Browser: year 5 update. *Nucleic acids research*. 41:D56-63.
6. Kumar S, Sanderford M, Gray VE, Ye J, Liu L. (2012) Evolutionary Diagnosis Method for Variants in Personal Exomes. *Nature Methods.* 9(9):855-6
7. Dubey R, Zhou J, Wang Y, Thompson PM, Ye J (2014) Analysis of sampling techniques for imbalanced data: An n = 648 ADNI study. *Neuroimage*. 15;87:220-41
8. Meinshausen N, Buhlmann P. (2010) Stability selection. *Journal of the Royal Statistical Society: Series B*. 72:417-73.
9. Liu J, Ji S, Ye J. (2009) SLEP: Sparse Learning with Efficient Projections. Arizona State University. http://www.public.asu.edu/~jye02/Software/SLEP

Sub-challenge #1, Rank #2 Model

Stepanov Oleg, team "OS"
Institute for Systems Biology Moscow.
ollegstepanov@gmail.com

Introduction

My approach was to develop a General Linear Model (GLM) class of base learner, using bootstrapping for model training and repeated random sub-sampling for validation. The use of a GLM framework was motivated by both its effectiveness and easy interpretation. Since the amount of data available was limited, I used bootstrapping for parameter selection and validation of my model.

I approached this problem without the use of any prior biological or medical knowledge, allowing the model to discern which features and feature combinations are most informative. In particular, I was interested in testing combinations and interactions of features using both RPPA data and clinical data.

Method

To pre-process the data, I first excluded clinical covariates that contained many "NA" values. While that could lead to information loss, I decided to use only parameters that contain sufficient information.

Model construction consists of several steps:

1) I created a list with all parameters and all possible linear combination of 2 and 3 parameters.

2) I checked the prediction ability for those models. I found that even if model consisting of one parameter has low prediction ability, some combination with that parameter could still have good predictive ability.

3) For each model, I split the initial data set into 2 parts and increased number of patients with bootstrapping. In general I had increased number of patients with CR and Resistant status to 1000 each by sampling with replacement. I repeated this step 1000 times and calculated the median BAC score for each model.

4) Next I sorted all the models by BAC score and selected all models with scores more than 0.5.

5) Finally, I found the best combinations of features from models with high scores. I started from best model and added successive models with lower scores. Analysis of predictive power was the same. If new model has higher score it becomes best model.

Conclusion
Using GLM method with multiple linear combinations yielded favorable results with the benefit of having a final model that is easy to interpret. One disadvantage was that this method required a great deals of computational time and power.

Sub-challenge #2, Rank #1 Model

A bagged semi-parametric model for predicting remission duration in AML sub-challenge 2

Xihui Lin : Eric.Lin@oicr.on.ca
Gregory M. Chen : gregorymchen@gmail.com
Honglei Xie : xhonglei2007@gmail.com
Geoffrey A. M. Hunter : Geoffrey.Hunter@oicr.on.ca
Paul C. Boutros : Paul.Boutros@oicr.on.ca

October 13, 2015

Summary

We implemented a bootstrap aggregated (bagged) Cox-like semi-parametric model with carefully selected predictors as a predictive model for remission duration for the AML sub-challenge 2.

1 Introduction

Why a bagged semi-parametric model

- The performance of the benchmark model using a standard Cox model with five selected clinical predictors is comparable to the top models on the leaderboard and better than our previous models such as survival random forests, boosted quantile regression, and weighted linear model.
- A native average over all submissions in sub-challenge 2 & 3 performs surprisingly well on the leaderboard, so we reasoned that averaging (via bagging) would be a viable strategy to reduce the variance.

Variable selection

- The benchmark model, with an impressive performance, uses only five selected clinical variables.
- From our work on sub-challenge 1, our model performance decreased whenever we incorporated one or more of the protein data, so we omitted this data from our models for sub-challenge 2 & 3 model.
- Based on these observations we started with the selected five clinical variables, i.e., *Age.at.Dx, Chemo.Simplest, HGB, ALBUMIN* and *cyto.cat*.

Missing values

Since there are only a few missing values of the selected variables in the training and testing sets, we simply replace them by the medians and modes for continuous and discrete variables respectively.

2 Methods

2.1 Preprocessing: re-categorizing 'cyto.cat'

This part was done and shown in the supplement for sub-challenge 3, using overall survival time as outcome. We didn't re-do the analysis for remission duration as the results were shown in [1] to be similar. The re-categorized *cyto.cat* is shown in Table 1.

Risk Category	Abnormality
High	'-5,-7', '-5,-7,+8', '-7', '-7,+8'
Intermediate (baseline)	'-5', '11q23', '8', 'IM', 'Misc', 't6;9', 't9;22', 'inv9'
Intermediate-low	'diploid'
Lower	'inv16', 't8;21'

Table 1: New cytogenetics categories.

2.2 Model

An appropriate model for bagging is one that should be unbiased with high variation. For this purpose, we added some 'frailty' terms (inspired by frailty in survival model and the positivity of the selected continuous variables) to the standard Cox proportional hazard model as

$$\lambda(t|x,z) \;=\; \lambda_0(t)x^{\alpha}\exp(x\beta + z\gamma) \tag{1}$$

where x is a vector of positive continuous variates and z is a vector of discrete variables, and

$$x^{\alpha} := (x_1, \cdots, x_k)^{(\alpha_1,\cdots,\alpha_k)} := \prod_{i=1}^{k} x_i^{\alpha_i}.$$

Indeed, equation (1) is equivalent to

$$\lambda(t|x,z) \;=\; \lambda_0(t)\exp((\log x)\alpha + x\beta + z\gamma). \tag{2}$$

We draw $B = 1000$ bootstrap samples. With each bootstrap sample, model (2) is fitted with R function *coxph*(R-3.1.1, survival-2.37-7). The final prediction from this bagged model is a simple average over all $B = 1000$ base models. Out-of-bag (OOB) samples are used to assess the performance, which typically slightly underestimates the performance. The OOB samples gives an estimate of 0.640 for Pearson correlation coefficient and 0.671 for C-index.

2.3 Quantiles for survival time (remission duration) prediction

The remaining question was to choose a survival time (remission duration) for prediction. A typical choice is the median survival time. However, Figure 1 shows that percentiles in interval $[0.1, 0.2]$ seem to be better choices. Again, motivated by model averaging, we chose to average survival quantiles over this interval as the final prediction. The performances, shown by the circled points on the right-most, look better than any single quantile.

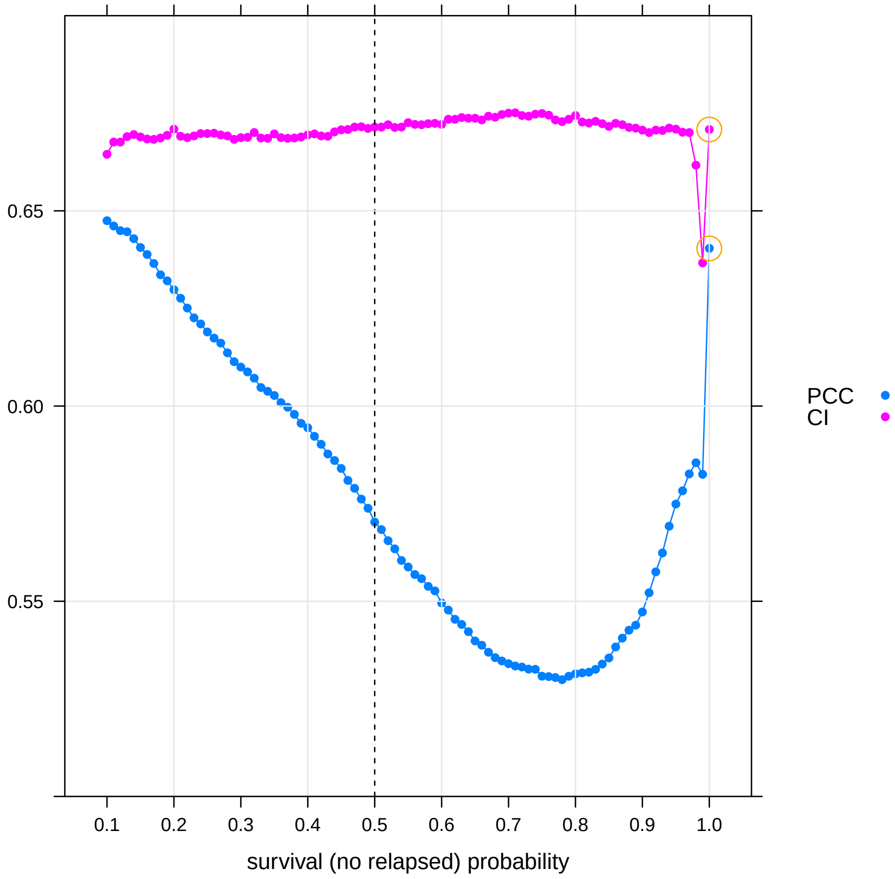

Figure 1: C-index (CI) and Pearson correlation coefficient (PCC) versus chosen survival (no relapsed) probability. The two circled points on the right-most side represent the performance of estimate by averaging over survival probability interval of $[0.1, 0.2]$.

3 Conclusion and Discussion

- The idea of averaging seems to give a better predictive model, though it generally produces a model hard to interpret.
- Our model used five clinical variables, *Age.at.Dx*, *Chemo.Simplest*, *HGB*, *ALBUMIN* and the re-categorized *cyto.cat*. Including additional clinical and/or proteomic variables, de-

termined by using a minimal-depth, survival random forest model [3], did not substantially improve model performance.

- Interactions and quadratic effects of the five selected variables were added to the model, but no significant improvement was observed.

References

[1] Grimwade D, Walker H, Oliver F, Wheatley K, Harrison C, Harrison G, Rees J, Hann I, Stevens R, Burnett A, Goldstone A (1998). The importance of diagnostic cytogenetics on outcome in AML: analysis of 1,612 patients entered into the MRC AML 10 trial. The Medical Research Council Adult and Children's Leukaemia Working Parties. *Blood*, 92 (7): 2322-33.

[2] Bennett JM, Catovsky D, Daniel MT, Flandrin G, Galton DA, Gralnick HR, Sultan C (1976). Proposals for the classification of the acute leukaemias. French-American-British (FAB) co-operative group. *Br J Haematol* 33 (4): 45-8. doi:10.1111/j.1365-2141.1976.tb03563.x

[3] Ishwaran H., Kogalur U.B., Chen X. and Minn A.J. (2011). Random survival forests for high-dimensional data. *Stat. Anal. Data Mining*, 4, 115-132

[4] Acute Myeloid Leukemia Outcome Prediction Challenge (syn2455683)

Authors Statement

- **Xihui Lin (Eric)**: Model development, validation and discussion; model and algorithm consultant; manuscript writeup.
- **Gregory M. Chen**: Model development, validation and discussion, and manuscript review.
- **Honglei Xie**: Model development, validation and discussion, and manuscript review.
- **Geoffrey A. M. Hunter**: Scientific oversight and discussion, and manuscript review.
- **Paul C. Boutros**: Scientific oversight, funding support, and manuscript review

Corresponding Author: Xihui Lin, Eric.Lin@oicr.on.ca

Sub-challenge #3, Rank #1 Model

A bagged semi-parametric model for predicting overall survival time in AML sub-challenge 3

Xihui Lin : Eric.Lin@oicr.on.ca
Gregory M. Chen : gregorymchen@gmail.com
Honglei Xie : xhonglei2007@gmail.com
Geoffrey A. M. Hunter : Geoffrey.Hunter@oicr.on.ca
Paul C. Boutros : Paul.Boutros@oicr.on.ca

October 13, 2015

Summary

We implemented a bootstrap aggregated (bagged) Cox-like semi-parametric model with carefully selected predictors as a predictive model for overall survival time for sub-challenges 3.

1 Introduction

Why a bagged semi-parametric model

- The performance of the benchmark model using a standard Cox model with five selected clinical predictors is comparable to the top models on the leaderboard and better than our previous models such as survival random forests, boosted quantile regression, and weighted linear model.
- A native average over all submissions in sub-challenge 2 & 3 performs surprisingly well on the leaderboard, so we reasoned that averaging (via bagging) would be a viable strategy to reduce the variance.

Variable selection

- The benchmark model, with an impressive performance, uses only five selected clinical variables.
- From our work on sub-challenge 1, our model performance decreased whenever we incorporated one or more of the protein data, so we omitted this data from our models for sub-challenge 2 & 3 model.
- Based on these observations we started with the selected five clinical variables, i.e., *Age.at.Dx, Chemo.Simplest, HGB, ALBUMIN* and *cyto.cat*.

Missing values

Since there are only a few missing values of the selected variables in the training and testing sets, we simply replace them by the medians and modes for continuous and discrete variables respectively.

2 Methods

2.1 Preprocessing: re-categorizing 'cyto.cat'

Cytogenetics (*cyto.cat* in AML dataset) is the single most prognostic factor in AML [1]. The distribution of patients across all cytogenetics categories in the AML data is imbalanced. This will negatively affect the performance of a bootstrapped model because some levels might be missing in a bootstrapped sample. Hence, we resolved to re-categorize the cytogenetics category into fewer, more balanced levels.

category	-5	-5,-7	-5,-7,+8	-7	11q23	21	8
frequency	4	8	3	5	7	2	12
category	IM	Misc	diploid	inv16	inv9	t6;9	t8;21
frequency	3	33	90	11	1	2	10

Table 1: Frequencies of levels of *cyto.cat in training set.*

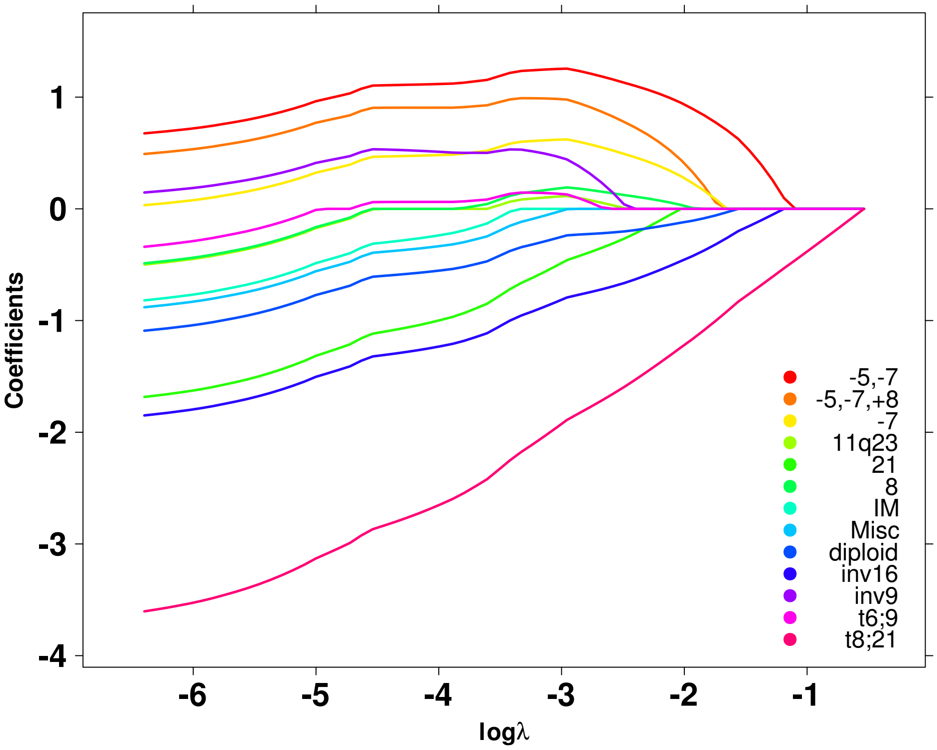

Figure 1: Coefficients vs $\log\lambda$. The numbers inside the figure indicating the index number of levels as in the same order in Table 1, with level '-5' chosen to be the baseline. So '-5' indicates level '-5,-7' and '13' indicats 't8;21'. Figure is plotted using BPG [4].

To this end, we fit a Cox model using 'cyto.cat' with an elastic-net penalty (R package *glmnet-1.9.8*). We set $\alpha = 0.5$ (the elastic-net penalty) in

$$\lambda(\alpha|\beta| + (1-\alpha)|\beta|^2/2).$$

Other values of α produced similar shrinkage paths to that in Figure 1. Based on Figure 1, we re-categorized 'cyto.cat' into 4 categories in Table 2. The 'diploid' category remains the same because it is the most abundant. The 't6;9' category, which is not in the training data, is grouped with 't9;22' for similarity. The 'inv9' category is grouped in the baseline category with all other chromosome 9 abnormalities because there is only one sample with this category in the training data. Lastly, the level '-7,+8' in the test sets is thought to be similar to '-5,-7,+8' and is consistent with the cytogenetic categories in Grimwade *et al.* [1] and Bennett [2].

Risk Category	Abnormality	Counts
High	'-5,-7', '-5,-7,+8', '-7', '-7,+8'	16
Intermediate (baseline)	'-5', '11q23', '8', 'IM', 'Misc', 't6;9', 't9;22', 'inv9'	62
Intermediate-low	'diploid'	90
Lower	'inv16', 't8;21'	21

Table 2: New cytogenetics categories.

2.2 Model

An appropriate model for bagging is one that should be unbiased with high variation. For this purpose, we added some 'frailty' terms (inspired by frailty in survival model and the positivity of the selected continuous variables) to the standard Cox proportional hazard model as

$$\lambda(t|x,z) \;=\; \lambda_0(t)x^{\alpha}\exp(x\beta + z\gamma) \tag{1}$$

where x is a vector of positive continuous variates and z is a vector of discrete variables, and

$$x^{\alpha} := (x_1, \cdots, x_k)^{(\alpha_1,\cdots,\alpha_k)} := \prod_{i=1}^{k} x_i^{\alpha_i}.$$

Indeed, equation (1) is equivalent to

$$\lambda(t|x,z) \;=\; \lambda_0(t)\exp((\log x)\alpha + x\beta + z\gamma). \tag{2}$$

We draw $B = 500$ bootstrap samples. With each bootstrap sample, model (2) is fitted with R function *coxph* (R-3.1.1, survival-2.37-7). The final prediction from this bagged model is a simple average over all $B = 500$ base models. Out-of-bag (OOB) samples are used to assess the performance, which typically slightly underestimates the performance. The OOB samples gives an estimate of 0.618 for Pearson correlation coefficient and 0.678 for C-index. Similar models with interaction and quadratic terms did not improve model performance.

2.3 Quantiles for survival time prediction

The remaining question was to choose a survival time for prediction. Typically, the median survival time is used, however, percentiles in interval $[0.1, 0.3]$ seem to be better choices (Figure 2). Again, motivated by model averaging, we chose to average survival quantiles over this interval for the final prediction. The performance, shown by the horizontal lines in Figure 2 is better than any single quantile.

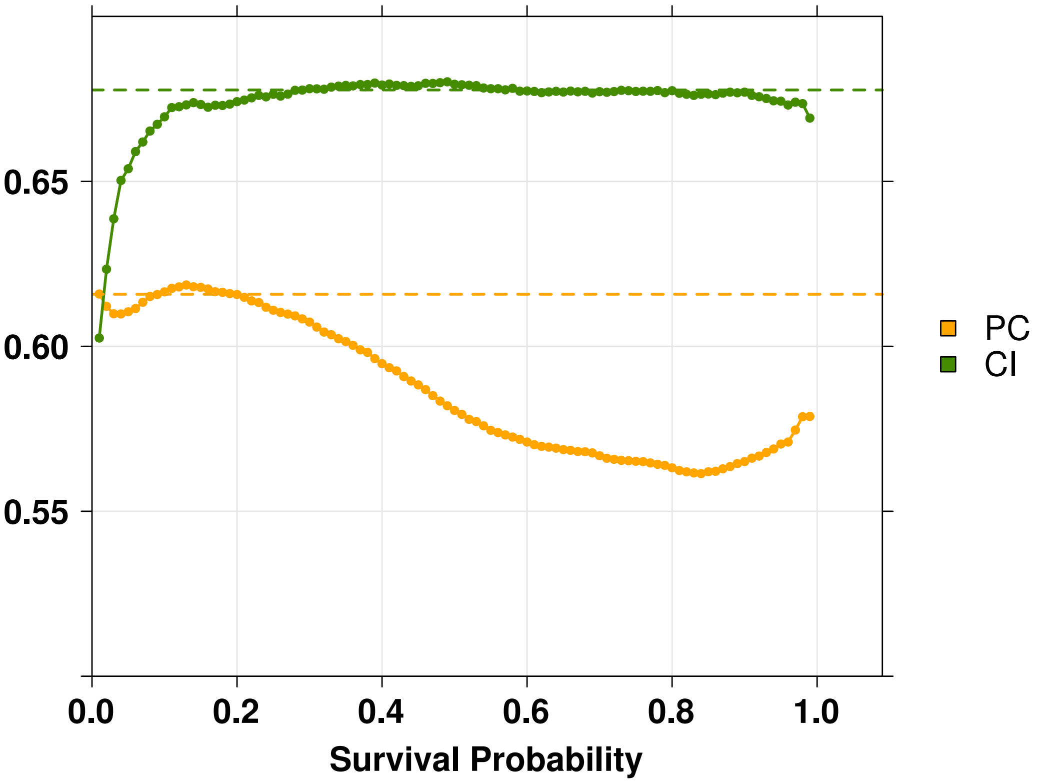

Figure 2: C-index (CI) and Pearson correlation coefficient (PCC) versus chosen survival probability. The horizontal lines represent the performance of estimate by averaging over survival probability interval of $[0.1, 0.3]$. Figure is plotted using BPG [4].

3 Experimental design: which changes improve performance?

Next, we wanted to assess the contribution of each modelling decision (bagging, adding log-transform of continuous variables, and averaging over survival quantiles) to the overall performance of the model. To this end, we performed a 10 fold cross validation with 10 repetitions on all model permutations as outlined in Table 3. The results, shown in Table 4, reveal that all the three model components improved PCC, especially using the mean survival quantiles whereas only bagging significantly improved the CI.

4 Conclusion and Discussion

- Averaging seems to improve model performance at the cost of interpretability.
- Our model used five clinical variables, *Age.at.Dx, Chemo.Simplest, HGB, ALBUMIN* and the re-categorized *cyto.cat*. Including additional clinical and/or proteomic variables, determined by using a minimal-depth, survival random forest model [3], did not substantially improve model performance.
- Interactions and quadratic effects of the five selected variables were added to the model, but no significant improvement was observed.

Bagging	Log Transform	Mean Survival	PCC	CI
0	0	0	0.5988	0.6857
0	0	1	0.6138	0.6868
0	1	0	0.6459	0.6622
0	1	1	0.6534	0.6588
1	0	0	0.6153	0.6969
1	0	1	0.6303	0.7011
1	1	0	0.6538	0.6982
1	1	1	0.6578	0.6990

Table 3: Result of a repeated cross-validation.

		Effect	p-value
PCC	Bagging	0.0113	0.0325
	Log Transform	0.0104	0.0423
	Mean Survival	0.0382	0.0004
CI	Bagging	0.0254	0.0172
	Log Transform	0.0007	0.9221
	Mean Survival	-0.0131	0.1138

Table 4: Result from linear regression on Pearson's Correlation Coefficient(PCC) and Concordance Index (CI).

- Overall, bagging improves both the PCC and CI, whereas the log-transformed terms and 'averaging over survival quantile' only improves the PCC.

References

[1] Grimwade D, Walker H, Oliver F, Wheatley K, Harrison C, Harrison G, Rees J, Hann I, Stevens R, Burnett A, Goldstone A (1998). The importance of diagnostic cytogenetics on outcome in AML: analysis of 1,612 patients entered into the MRC AML 10 trial. The Medical Research Council Adult and Children's Leukaemia Working Parties. *Blood*, 92 (7): 2322-33.

[2] Bennett JM, Catovsky D, Daniel MT, Flandrin G, Galton DA, Gralnick HR, Sultan C (1976). Proposals for the classification of the acute leukaemias. French-American-British (FAB) co-operative group. *Br J Haematol* 33 (4): 45-8. doi:10.1111/j.1365-2141.1976.tb03563.x

[3] Ishwaran H., Kogalur U.B., Chen X. and Minn A.J. (2011). Random survival forests for high-dimensional data. *Stat. Anal. Data Mining*, 4, 115-132

[4] P'ng et al. (submitted, 2015), BPG: a package to visualize scientific data. *http://labs.oicr.on.ca/Boutros-lab/software/bpg*

[5] Acute Myeloid Leukemia Outcome Prediction Challenge (syn2455683)

Authors Statement

- **Xihui Lin (Eric)**: Model development, validation and discussion; model and algorithm consultant; manuscript writeup.
- **Gregory M. Chen**: Model development, validation and discussion, and manuscript review.
- **Honglei Xie**: Model development, validation and discussion, and manuscript review.
- **Geoffrey A. M. Hunter**: Scientific oversight and discussion, and manuscript review.
- **Paul C. Boutros**: Scientific oversight, funding support, and manuscript review

Corresponding Author: Xihui Lin, Eric.Lin@oicr.on.ca
