# Supplementary material for: A Crowdsourcing Approach to Developing and Assessing Prediction Algorithms for AML Prognosis
Source: PLoS Comput Biol. 2016 Jun 28;12(6):e1004890. doi: 10.1371/journal.pcbi.1004890 (PMC4924788; doi:10.1371/journal.pcbi.1004890)
Supplement: S2 Text — (PDF) [file pcbi.1004890.s010.pdf]

DREAM 9 AML-OPC Consortium

Team 89

Jose M. G. Vilar[1]

1 Biophysics Unit (CSIC-UPV/EHU) and Department of Biochemistry and Molecular Biology, University of the Basque Country, Bilbao, Spain

Team Attractor Metagenes

Tai-Hsien Ou Yang[1], Dimitris Anastassiou[1]

1 Department of Systems Biology and Department of Electrical Engineering, Columbia University, New York, NY

Team BostonCompBio

Reynald Lescarbeau, Rebecca Lescarbeau

Team Chipmunks.

Honglei Xie[1], Greg Chen[1], Xihui Lin[1], Geoffrey Hunter[1]

1 Ontario Institute for Cancer Research, Toronto, ON, Canada

Team Clinical Persona

Ljubomir Buturovic[1], Damjan Krstajic[1,2], Alejandrina Pattin[1]

1 Clinical Persona Inc, East Palo Alto, CA, USA

2 Research Centre for Cheminformatics, Beograd, Serbia

Team Creighton

Chad J. Creighton[1,2]

1 Department of Medicine and Dan L. Duncan Cancer Center, Baylor College of Medicine, Houston, TX, USA

2 Department of Bioinformatics and Computational Biology, The University of Texas M. D. Anderson Cancer Center, Houston, TX, USA

Team CS

Sunho Park[1], Minsoo Kim[1], Alan Estrada[1], Jae-Ho Cheong[2,3], Tae Hyun Hwang[1]

1 Department of Clinical Sciences, University of Texas Southwestern Medical Center, Dallas, TX, USA

2 Department of Surgery, Yonsei University College of Medicine, Seoul, South Korea

3 Department of Biochemistry & Molecular Biology, Yonsei University College of Medicine, Seoul, South Korea

Team EPD

Sunil Kumar[1,2], René Dreos[2], Giovanna Anbrosini[1,2], Philipp Bucher[1,2]

1 The Swiss Institute for Experimental Cancer Research (ISREC), School of Life Sciences, EPFL, Lausanne, Switzerland

2 The Swiss Institute of Bioinformatics (SIB), EPFL, Lausanne, Switzerland

Team EvoMed

Li Liu[1]

1 Department of Biomedical Informatics, Arizona State University, Tempe, AZ, USA

Team ICM CUDA Dreamers

Paweł Żuk[1], Antoni Rościszewski[1], Rafał Niemiec[2]. Mariusz Wrzesień[2], Witold Rudnicki[1]

1 Interdisciplinary Centre for Mathematical and Computational Modelling, University of Warsaw, Warsaw, Poland

2 University of Information Technology and Management in Rzeszów, Rzeszów, Poland

Team JamesMcM

James McMurray[1]

1 Max Planck Institute for Intelligent Systems, Tübingen, Germany

Team JP

Jinpu Li[1]

1 University of Science and Technology of China, Hefei, Anhui, China

Team JTNL

Tom Ronan[1], Jennifer Flynn[2], Kristen Naegle[1]

1 Department of Biomedical Engineering, Washington University in St. Louis, St. Louis, MO, USA

2 Division of Biology and Biomedical Sciences, Washington University in St. Louis, St. Louis, MO, USA

Team Jyothi

Jyothi Korra[1]

1 Christ University, Bengaluru, Kanataka, India

Team KAATZ-at-OSU

Kelly Regan[1], Tasneem Motiwala[1], Daniel Morgan[1], Zachary Abrams[1], Andrea Peabody[1], Andrew Fitzgerald[1]

1 Department of Biomedical Informatics, The Ohio State University College of Medicine, Columbus, OH, USA

Team nadal

Daniel Kim

Team Naoned e Breizh

Loic Campion[1,2]

1 Integrated Center of Oncology, Biometrics Unit, St-Herblain, France

2 Cancer Research Center Nantes-Angers, Nantes, France

Team sg_139

Subharup Guha[1], Veerabhadran Baladandayuthapani[2,3]

1 Department of Statistics, University of Missouri, Columbia, MO, USA

2 Department of Biostatistics, The University of Texas MD Anderson Cancer Center, Houston, TX, USA

3 Department of Biostatistics, Rice University, Houston, TX, USA

Team Snail

Tiziana Sanavia[1], Barbara Di Camillo[1]

1 Department of Information Engineering, University of Padova, Padova, Italy

Team ssss

Seyyed A. Fatemi[1], Anthony Kuh[1]

1 Electrical Engineering Department, University of Hawaii at Manoa, Honolulu, HI, USA

Team SWlab

Kimberly Batten[1], Jerry Shay[1], Woodring Wright[1]

1 UT Southwestern Medical Center at Dallas, Dallas, TX, USA

Team SWP

Scott W. Piraino

Team test_ho

Maryam Hosseini[1], Narayana Santhanam[1]

1 Electrical Engineering Department, University of Hawaii at Manoa, Honolulu, HI, USA

Team TT

Hao Tang[1], Xiao Zang[1], Tao Wang[1], Guanghua Xiao[1], Yang Xie[1]

1 University of Texas, Southwestern Medical Center, Dallas, TX, USA

Team YL

Yashu Liu[1], Jieping Ye[1], Sen Yang[1], Tao Yang[1]

1 Arizona State University, Tempe, AZ, USA

Team yoda

Maciej Fronczuk[1], Weiyi Gu[1], Ling-Hong Hung[1], Vivian G. Oehler[2], Kiyana Zolfaghar[1], Ka Yee Yeung[1]

1 Center for Data Science, Institute of Technology, University of Washington, Tacoma, WA, USA

2 Clinical Research Division, Fred Hutchinson Cancer Research Center, Seattle, WA, USA
